# Life-Cycle Assessment and Environmental Costs of Cement-Based Materials Manufactured with Mixed Recycled Aggregate and Biomass Ash

**DOI:** 10.3390/ma17174357

**Published:** 2024-09-03

**Authors:** Francisco Agrela, Manuel Rosales, Mónica López Alonso, Javier Ordóñez, Gloria M. Cuenca-Moyano

**Affiliations:** 1 Construction Engineering Area, University of Córdoba, 14071 Córdoba, Spain; z02rosgm@uco.es; 2 Civil Engineering School, University of Granada, 18001 Granada, Spain; mlopeza@ugr.es (M.L.A.); javiord@ugr.es (J.O.); 3 Department of Building Constructions, University of Granada, 18001 Granada, Spain; gloriacuenca@ugr.es

**Keywords:** life-cycle assessment, life-cycle cost, mixed recycled aggregates, biomass bottom ash, mortar, concrete

## Abstract

The development of new building elements, such as concrete and mortar with sustainable materials, which produce a lower carbon footprint, is an achievable milestone in the short term. The need to reduce the environmental impact of the production of cement-based materials is of vital importance. This work focuses on the evaluation of the life-cycle assessment, production costs, mechanical performance, and durability of three mortars and three concrete mixtures in which mixed recycled aggregates (MRAs) and biomass bottom ash from olive waste (oBBA) were included to replace cement and aggregates. Powdered MRA and oBBA were also applied as complementary cementitious materials with a reduced environmental footprint. Chemical and physical tests were performed on the materials, and mechanical performance properties, life-cycle assessment, and life-cycle cost analysis were applied to demonstrate the technical and environmental benefits of using these materials in mortar and concrete mixtures. This research showed that the application of MRA and oBBA produced a small reduction in mechanical strength but a significant benefit in terms of life-cycle population and environmental costs. The results demonstrated that finding long-term mechanical strength decreases between 2.7% and 14% for mortar mixes and between 1.7% and 10.4% for concrete mixes. Although there were small reductions in mechanical performance, the savings in environmental and monetary terms make the feasibility of manufacturing these cement-based materials feasible and interesting for both society and the business world. CO_2_ emissions are reduced by 25% for mortar mixes and 12% for concrete mixes with recycled materials, and it is possible to reduce the cost per cubic meter of mortar production by 20%, and the savings in the cost of production of a cubic meter of concrete is 13.8%.

## 1. Introduction

The use of waste as a recycled product in the manufacture of concrete is a widespread practice. The use of natural processed materials, such as cement or natural aggregates, has a significant environmental impact, both in terms of CO_2_ emissions and the modification of ecosystems. Cement accounts for about 10% of the mass of concrete, 4 Gigatonnes per year, the same quantity as global food consumption [1]. Building-related activities generate 7.7 Gigatonnes of CO_2_ per year, of which cement generates 36% [2]. The processes that emit the most CO_2_ are hydrocarbons for the calcination and decarbonation of limestone, related to the production of clinker [3,4]. With respect to aggregates, 17.5 Gigatonnes per year are included as gravel and sand in the production of concrete [5].

As cement is the most environmentally costly element of the building material, the use of supplementary cementitious materials (SCMs) is of vital importance. Elements, such as fly ash, blast-furnace slag [6], Metakaolin, or silica fume, replace part of the cement used in a concrete mix.

By increasing the degree of substitution and optimising mixtures, a 45% reduction in CO_2_ emissions can be achieved [3]. In addition, there are recycled materials, such as Construction & Demolition Waste (CDW) [7], with a high clay and Biomass Bottom Ash (BBA) [8] composition that can be included as cement substitutes with a previous crushing process, without including a calcining process. There are studies in which recycled aggregates are included in cementitious matrices. The study carried out by Chen et al. [9] includes mechanical resistance tests in which the concrete has been reinforced with steel and plastic fibers. Furthermore, this study determines the behaviour under eccentric loads, bringing us closer to the real usefulness of these materials. In addition, the study carried out by Teng et al. [10] includes basalt fibre reinforced polymer bars and reinforced geopolymer concrete being subjected to seawater in terms of corrosion and durability.

Currently, there is a diversity of regulations and uses of recycled elements in concrete for buildings depending on the country of focus. In Spain, mixed recycled aggregate is limited to 20% as a substitute for coarse aggregate [11], although the scientific literature determines that the use of recycled elements in concrete can be feasible in higher percentages and as a substitute for cement or fine aggregates [12]. Both in Spain and in other countries, there is reluctance, materialised through regulations, to use recycled elements in structural concrete. There are studies that have evaluated the use of recycled aggregate for columns and beams as reinforced concrete, finding results of mechanical properties and durability comparable to conventional concrete. Failures and cracks are found in concentric and eccentric loads with the same progression as conventional concrete [13].

Other materials, such as calcined clay [14], present better values, but, as the calcining temperature increases to 900 °C, the pozzolanicity decreases, with the fall in resistance being more accentuated. Clays calcined at 600 °C show a high specific surface area and the complete dehydroxylation of kaolinite but incomplete decomposition of the clay. For temperatures of 800 °C, it continues to show an adequate specific surface and a partial decomposition of the clay, being in an adequate structural disorder. However, when calcination temperatures exceed 900 °C, we find that crystals form, decreasing their specific surface and finding limitations in their hardening via hydration [15].

Life-cycle analysis (LCA) is the collection and evaluation of the inputs, outputs, and potential environmental impacts of a product system throughout its life cycle. This methodology makes it possible to quantify the associated environmental loads, as well as to identify the processes that contribute significantly to the impact, which is why it is a fundamental application tool in the design of materials, products, or systems [16,17].

In this regard, LCA has been used in numerous studies to assess the environmental impact associated with the manufacture of construction and building materials, such as concrete [18,19,20,21,22,23], ceramic tiles [24], thermal insulation materials [25], expanded clay [26], phase change materials [27], wood-based building materials [18,28], or cement [29,30,31,32,33].

Likewise, in the current context of the circular economy, the manufacture of construction and building materials with secondary materials from waste entails an environmental benefit that can be analysed and quantified through the application of LCA. Consequently, research has been carried out that has technically and environmentally evaluated the incorporation of recycled aggregates from construction and demolition waste or biomass ash as a replacement for natural aggregates or cement in the manufacture of building materials, such as masonry mortar [34,35], concrete [19,36], precast concrete [37,38], ceramic tiles [24,39,40], bricks [41,42], road pavement [43], and warm mixture asphalt [44] These studies have determined the reduction of environmental loads associated with the substitution of raw materials for secondary materials, which also avoids the final disposal of waste in the landfill [45,46,47].

Environmental and Material Costs (EMCs) are the total costs of a construction applied to their parts throughout their life, including the costs of planning, design, construction, operations, maintenance, and disposal, minus any residual value. EMCs can address a period of analysis, which covers the entire life cycle, or selected stage(s) or periods of interest therein.

Including an EMC analysis in mortar and concrete mixture studies together with LCA, could be very useful for decision makers to choose solutions that are more efficient from an economic and environmental point of view, and even more so with the increase in construction, maintenance, and rehabilitation costs. The use of EMCs as a tool nowadays is needed to solve several problems, as pointed out in the literature, such as selecting the correct discount rate and agency costs or quantifying non-agency costs as user costs [48].

In recent years, some experiments have been carried out with the aim of reducing the environmental impact of mortar and concrete, substituting part of the natural materials for recycled aggregates. The integrated use of LCA and EMCs could facilitate the decision to choose the most sustainable option [49,50]. In this research, the authors analyse the costs of the different materials used to manufacture mortar and concrete mixtures that replace natural aggregates with recycled aggregates in order to assess which is the most economical solution.

This study carries out a study of the mechanical behaviour, environmental impact, and monetary savings of different concrete and mortar mixes with recycled materials. Firstly, a description and characterisation of the materials in this crude material is established, followed by a study of the mechanical behaviour and durability of the cement-based mixes studied. Once the technical feasibility has been demonstrated, the basis for an environmental study is established by means of software that calculates the pollution savings of the recycled mixes. Next, the mortar and concrete mixes studied are studied in economic terms and can be compared with conventional mixes.

It is important to quantify the positive aspects of introducing recycled materials into cement-based mixes. One of the most appropriate ways to make the use of recycled materials attractive is to show the pollution savings and savings in economic terms. This study brings together mixed research, ranging from the technical aspects of cement-based materials (mechanical strength and durability) to environmental and monetary research, studying the LCA and EMCs of different mortar and concrete mixtures. The transfer of knowledge is essential in scientific activity, so quantifying monetary and environmental savings by establishing the technical feasibility is an important objective in scientific progress, making the production of cement-based materials with recycled materials attractive.

## 2. Materials and Methods

The building materials included in this work were divided into two types, materials applied in mortar mixtures and materials applied in concrete mixtures. Recycled materials were included as supplementary cement materials (pBBA and pMRA) and materials replacing natural aggregates (fine MRA for mortar mixtures and BBA and MRA for concrete mixtures) and conventional materials (standard natural sand, ordinary Portland cement, and natural aggregates). Also included in this section are the dosages of mortar and concrete. Physicochemical analyses were carried out for all [51].

### 2.1. Mortar Component Materials

#### 2.1.1. Cement CEM I 42.5R

The cement used in this research was a commercial CEM I 52.5R (Votorantim cements, Málaga, Spain), and the chemical composition is summarised in Table 1.

#### 2.1.2. Powder of Biomass Bottom Ash (pBBA)

With respect to the replacement of cement with waste, biomass bottom ash powder is presented in this section. This material comes from Linares, Andalucía, Spain, where the power generation plant is located and where the fuel used is olive pruning and olive cake derived from oil extraction. The ash was milled to a grain size between 0 mm and 0.125 mm, obtaining pBBA. The properties of the powder pBBA derived from the physical and chemical characterisation are presented in Table 2.

The density is lower than cement, 2840 kg/m^3^, and the chemical composition shown based on X-ray fluorescence determines that the main elements that make up this powder are SiO_2_, CaO, K_2_O, and Al_2_O_3_. These component values are similar to those in other studies. The amounts of SiO_2_ (28%), CaO (30%), and Al_2_O_3_ (4%) are similar to the study carried out by Carrasco-Hurtado et al. [57]. With respect to K_2_O, the hydration of alkalis, such as potassium, causes changes in rheological properties, setting the time and mechanical strength. The hydration of alkalis, such as this element, causes instability in dimensional changes and alters the internal structure of cement-based materials [58].

#### 2.1.3. Processed Mixed Recycled Aggregates (pMRA)

The recycled aggregate is produced by GECORSA, a construction and demolition waste company, whose plant is located in Córdoba, Andalucía, Spain. The material was used to replace both cement and aggregate. This section presents the characteristics of the powder obtained for cement substitution, which is obtained by crushing and sieving until a grain size between 0 mm and 0.125 mm is obtained. Table 3 shows the data obtained from the characterisation of the powder from the pulverised recycled aggregate.

The density of MRA powder is lower than that of the cement used, CEM I 42.5R. Observing the elemental composition, similarities were found with other studies, such as the one published by Medina et al. [59]. They found that powders with high Al_2_O_3_ and Fe_2_O_3_ contents give competent pozzolanic and mechanical strength results. The chloride and sulphate contents are an indication of the quality of this supplementary cement material. Regulations determine the limits of chloride and sulphate content values, these being 0.1% and 4%, respectively [7]. The content of alkaline elements indicates the durability of cement-based materials, such as mortar and concrete mixtures [60]. MgO and K_2_O content results shows low values of 2.83% and 1.93%, respectively.

#### 2.1.4. Normalised Sand (SNS) 0/2 mm Size According to EN 196-1 [61]

Natural sand used in the manufacture of mortar mixtures is standardised according to EN 196-1 [61]. It is a siliceous sand, and the particle distribution is determined in Table 4. This sand is artificially produced by sieving in several steps until the desired grain size is achieved.

#### 2.1.5. Mixed Recycled Fine Aggregates (Fine MRA) 0/2 Size, from Gecorsa Company (Córdoba, Spain)

The mixed recycled aggregates with grain size 0/2 used to replace sand in mortar mixtures comes from GECORSA. To obtain this grain size, it was screened by eliminating the fraction above 2 mm. It was the same type of aggregates from which pMRA was obtained for cement substitution.

The gross composition, according to UNE-EN 933-11 [61], is shown in Table 5. The quality of recycled aggregates was able to be defined using this test. These results define that the recycled mixed aggregates used were of high quality.

It can be observed that it was mainly composed of concrete aggregates and natural aggregates, and these recycled aggregates are considered to be of quality, without impurities, such as soil or metals. These results show that the aggregates can provide a good mechanical behaviour for mortar and concrete mixtures.

Table 6 shows a summary of the physical qualities and chemical properties that define GECORSA’s MRA. All tests were performed under the standards described below.

Two of the most important values for the design of mortar and concrete mixtures are density and water absorption. Density, being different from a natural aggregate, causes the volume occupied for the same mass to be different. It is necessary to adjust the weights so that the volume is conserved. Regarding water absorption by the aggregate, it must be taken into account for the total amount of water to be added to each mix. If only the water established based on the water–cement ratio is added, the aggregates may absorb water, preventing the cement from fully hydrating. For this reason, saturation water is added, which is necessary to saturate the aggregate, leaving water available for the complete hydration of the cement. Other authors [64] broke down the regulatory limits of these values in his study. The most restrictive values for good mechanical performance determine that MRA density must be greater than 2.2 kg/dm^3^ and water absorption less than 7%. Since the MRA studied in this article has a high content of ceramic particles, the absorption of the 0–4 mm fraction is higher than this value. However, the granulometric fraction of 4–22 mm has an absorption lower than the most restrictive limit presented by Brito et al. [64].

The friability and sand equivalent values obtained in this study were 23.9% and 75.28%, respectively. With respect to physical parameters, three recycled aggregates with friability ratios between 24% and 27% have been studied [65]. Previous studies give higher values of friability and sand equivalents; for example, the fine recycled sand in one of these studies has a friability ratio of 32% [66]. The sand equivalent of this recycled aggregate is 86%.

Chemical parameters, such as chlorides and sulphates [67], compared the values established by Spanish regulations with values derived from a literature review. Spanish regulations establish that chlorides should be less than 0.05% and sulphates in water and acid less than 1% and 0.8%, respectively.

#### 2.1.6. Admixture

Mortar mixtures containing recycled materials show lower workability, making these mixtures not as fluid as control mixtures, so a commercial superplasticiser admixture, called SIKAMENT 3003 ES from the company SIKA (Madrid, Spain), was included in the mixtures with recycled substitutes.

Thanks to this admixture, the viscosity of the mixtures was achieved, matching the workability of the control mix. In addition, the amount of water was reduced, so that the water added was the amount necessary to hydrate the recycled phases and hydrate the cement. This point is important because an excess of water causes the mixtures to contain more porosity, making the mechanical strength lower than expected.

### 2.2. Component Materials of Concrete Mixtures

This section presents the materials used for concrete mixtures. It should be noted that the milled MRA and milled BBA powder is the same as that used for the mortar mixtures, so the characterisation is not presented again. Mixed recycled aggregates implemented in the concrete mixtures come from the same recycling plant, GECORSA, but in this case, we will focus on describing the parameters of the particle size fraction of 4–22 mm. The 0–4 mm fraction was described in the mortars section.

#### 2.2.1. Cement CEM II 42.5

The cements used in this research were CEM II 42.5 and CEM I 52.5. The physicochemical properties of CEM II 42.5 are summarised in Table 7, and this type of cement presented around a 18% of Limestone filler. The properties of CEM I 52.4 are summarised in Table 1.

The cement used was a type of cement with high compressive strength, especially suitable for mass concrete, reinforced concrete, non-prestressed prefabricated concrete, and for the manufacture of mortar mixtures in general.

#### 2.2.2. Natural Aggregates

**Figure 1:**
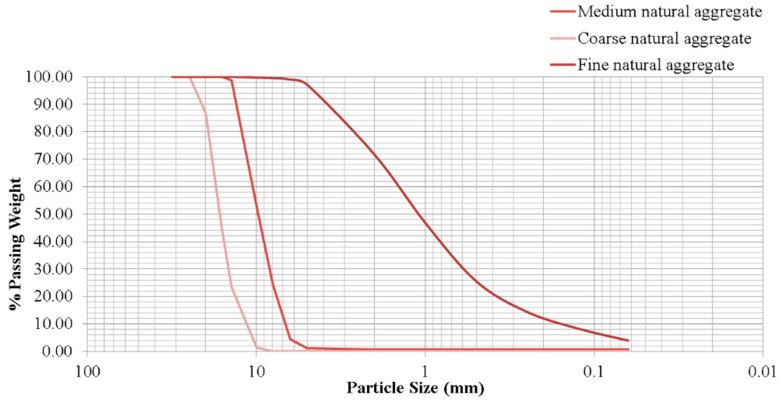
Particle size distribution of natural aggregates.

These aggregates came from a dolomitic quarry located in the municipality of Cordoba. The aggregates were divided into three parts depending on their grain size: fine natural aggregates (0–4 mm), medium natural aggregates (4–12 mm), and coarse natural aggregates (12–22 mm). Its physical characterisation is shown in Table 8 and particle size distribution in Figure 1.

Natural aggregates present a density and absorption in line with other works [70,71], where it establishes the density of medium and coarse natural aggregates (4–22 mm) at 2780 kg/m^3^ and 2650 kg/m^3^, respectively and, with respect to their absorption, 1.91% and 1.8%, respectively. Natural sand previously studied has a density of 2600 kg/m^3^ and water absorption of 0.6% [72]. This same study characterises natural aggregates of a grain size of 10–14 mm with a Los Angeles coefficient of 15%, a density of 2630 kg/m^3^, and water absorption of 0.3. A study analysing the properties of natural sands from different locations in Turkey presents three river sands. These sands have sand equivalents between 83% and 95% and densities between 2560 kg/m^3^ and 2620 kg/m^3^ [73]. Natural aggregates of siliceous nature from western Saudi Arabia have been studied. The Los Angeles coefficient and the ACV coefficient have values close to those of the natural aggregates studied in this paper. They obtained values from 14% to 24% for the angels and from 14% to 22% for the ACV [74].

#### 2.2.3. Mixed Recycled Aggregates (MRAs) (0/22 mm)

**Figure 2:**
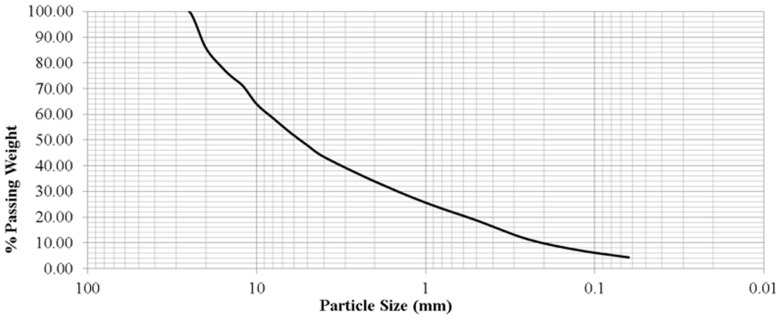
Particle size distribution of MRA.

To perform the characterisation of mixed recycled aggregates for concrete production the distinction between fine and coarse was made for the calculation of density and absorption. The Friability ratio, Sand equivalent, Aggregates Crushing Value, and Los Angeles coefficient were calculated following the standards. The results obtained of physical characterisation are presented in Table 9, with the particle size distribution in Figure 2.

Numerous studies have characterised mixed recycled aggregates for the manufacture of cement-based materials. Several mixed recycled aggregates have been studied, and their dry bulk density was between 2590 kg/m^3^ and 2670 kg/m^3^ [75]. The water absorptions presented by this study were between 5.42% and 10.05%. Another study characterised three types of fine recycled aggregates for the manufacture of mortar mixtures. In this study, the friability index of the recycled fine aggregates was between 24.02% and 27.20% [65], in accordance with the results obtained from the MRA studied in this paper. For the absorption of this recycled sand, the range of values was between 6.12% and 7.48%. Thirteen construction and demolition wastes from different parts of the Iberian Peninsula were studied [76]. Of the 13 materials studied, 9 had a coefficient of Los Angeles between 31.27% and 40.99%, values very close to GECORSA’s mixed recycled aggregate. Observing the results obtained from the characterisation of GECORSA’s mixed recycled aggregate, the low values of the Los Angeles coefficient and friability ratio, together with a high value of the sand equivalent in comparison with other mixed recycled aggregates, made this material feasible for the production of cement-based materials, such as concrete or mortar.

#### 2.2.4. Biomass Bottom Ash (BBA) (0/4 mm)

Biomass bottom ash is composed of unburned coarse particles produced in the primary combustion chamber during the biomass energy production process [77,78]. The BBA came from the biomass power plant located in Linares of the company Sacyr Industrial. The biomass used as fuel supply for electricity generation is composed of 60% wood, from almond and olive tree pruning, and 40% olive cake.

The physic-chemical properties of BBA are shown in Table 10. BBA shows a low bulk density value (1.94 kg/dm^3^) and a high-water absorption (19.82%). Researchers with a long history of studying this residue had previously tested biomass bottom ash for construction and building [77]. This paper studies ash from different power generation plants and at different moments of the year. The absorption values were between 27% and 14% and, for density values, between 1.82 kg/dm^3^ and 2.26 kg/dm^3^. However, friability ratios of this study were higher than the BBA included in this work (between 23% to 34%). Previous work on the Iberian Peninsula states that the amount of organic matter in biomass ash from olive pruning is 5.10% [79], somewhat higher than the ash under study. A more in-depth study of the biomass bottom ash from olive burning was carried out before [78]. In this study, the raw ash was found to have a sulphate content of 0.29 and 0.31 (water and acid soluble, respectively). Water absorption and density were also determined, with these values being 21.8% and 1860 kg/m^3^.

#### 2.2.5. Admixture

In concrete mixtures with recycled materials, as in mortar mixtures, a commercial superplasticiser admixture, called VISCOCRETE 6003 NG from the company SIKA, was included in the dosage. The main objective was to equalise the workability of the recycled mixtures to the control mixture without adding more water. When more water was added to the mix, taking into account the hydration water of the cement and the saturation water to hydrate the recycled elements, the mechanical strengths were reduced. By adding this superplasticising admixture, a viscosity of the concrete with recycled elements was achieved in accordance with the viscosity of the conventional reference concrete without reducing its mechanical capacities.

### 2.3. Dosages

Three mortar and three concrete mixtures were made to be evaluated in terms of environmental cost, monetary cost, and mechanical performance.

In the mortar mixtures, a control mortar made with CEM II 42.5, standard sand, and a water–cement ratio of 0.5 was included. In addition, two more mixtures were performed with CEM I 52.5. The first replaced cement with a powder mixture from MRA and BBA. Six percent of the cement was replaced by pBBA, and 19 percent of the cement was replaced by pMRA. Due to previous studies [8], the pozzolanic capacities of pMRA were higher than pBBA due to the high content of ceramic elements. The third mortar mixture studied in this work contained a cement substitution as the previous one (6% pBBA and 19% pMRA), in addition to substituting 20% of the normalised sand with the fine fraction of MRA (particle size between 0 and 2 mm). The dosages are broken down in Table 11.

By adding the substitution of 20% of natural normalised sand for the fine fraction of MRA, it found that the need to hydrate the aggregates resulted in the existence of absorption water. In addition, to preserve the consistency of the mortar mixtures, an admixture was included at 0.22% by weight of the cement. The consistency of the mortar mixtures was 20.7 cm.

In the concrete mixtures, a control mixture with conventional cement and natural aggregates was also included. In this case, the cement substitution was given with 19% pMRA and 6% pBBA for the same reason as explained above. In addition, in the third mix, natural aggregates were replaced with mixed recycled aggregates and biomass bottom ash. The concrete dosages are determined in Table 12.

All mixtures had a water–cement ratio of 0.42. Note that the densities of pMRA and pBBA were lower than that of cement. For dosages with cement replacement, the total weight of powders (cement, pMRA, and pBBA) was less for the same volume, so the water required to hydrate all phases was less. The saturation water required to achieve a good workability is specified in the saturation water column.

## 3. Experimental Methods and Results of Mechanical Behaviour of Mortars and Concrete Mixture

### 3.1. Compressive Strength and Flexural Strength in Mortar and Concrete Mixtures

To obtain the mechanical performance of each of the mixtures, the compressive strength of the concrete and mortar mixtures was studied. Compressive strength was determined according to UNE-EN 12390-3 [80] for concrete and according to UNE-EN 196-1 [61] for mortar mixtures. For the mortar and concrete mixtures, the mechanical compressive strengths at 7, 28, and 90 days were obtained. For each of the ages, 6 mortar specimens of 4 × 4 × 4 cm and 3 concrete specimens of 10 × 10 × 10 cm were broken, and the average values obtained were presented.

Also, flexural strength was determined according to UNE-EN 12390-5 [81] for concrete mixtures with 2 specimens and according to UNE-EN 196-1 [61] for mortar mixtures with 3 specimens. Average values are presented in this work. These mechanical performances were determined at 7, 28, and 90 days.

#### 3.1.1. Results of Compressive Strength and Flexural Strength of Mortar Mixtures

**Figure 3:**
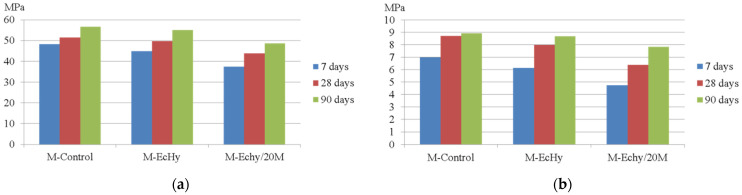
(**a**) Compressive strength for mortar mixtures; (**b**) flexural strength for mortar mixtures.

Mortar mixtures were tested under standardised compressive and flexural strength tests, and the results were obtained at 7, 28, and 90 days. The results are shown in Table 13 and Figure 3.

In the mortar mixtures that substituted cement and sand (M-Echy/20M), there was a more accentuated drop in strength with respect to the control mix. The drops in mechanical compressive strength were 22.5%, 14.8%, and 14.2%. This behaviour may be due to two factors: the higher porosity [82] of the mixtures with mixed recycled aggregates replacing sand and the pozzolanicity of the new additions [83], which are not as cementitious as clinker. In other studies, cement was replaced with BBA, a 20% replacement, having a lower mechanical performance, reaching 41.78 MPa [78]. Compressive strength was able to determine flexural strength. These values are in accordance with other studies [84,85].

#### 3.1.2. Results of Compressive Strength and Flexural Strength of Concrete Mixtures

**Figure 4:**
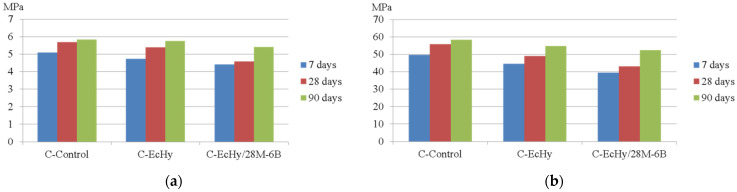
(**a**) Compressive strength for concrete mixtures; (**b**) flexural strength for concrete mixtures.

The concrete mixtures were tested in terms of mechanical strength and dimensional changes. The compressive and flexural strengths at 7, 28, and 90 days were performed according to standards. The results are shown in Table 14 and Figure 4.

In concrete mixtures with recycled elements, they cause a drop in strength with respect to the control mix. Although in the long term, the drops in compressive and flexural strength were smaller, with drops at 90 days of 6.3% for the C-EcHy mixture that replaced only cement and 10.4% for the C-EcHy/28M-6B mixture that replaced cement and aggregate. Other studies like [86] determined that the drop in compressive strength of concrete with cement and aggregates replacement with BBA and MRA at 90 days is 10% with respect to the control concrete. This percentage is in accordance with this study.

### 3.2. Dimensional Changes in Mortar and Concrete Mixtures

During the curing process, the dimensional changes that occurred in the mortar and concrete mixtures were measured. A micrometre precision comparator was used for these measurements. For the concrete, the specimens tested were 4 × 4 × 28.5 cm, and for the mortar mixtures, the specimens tested had original dimensions of 2 × 2 × 28.5 cm. In order to observe the evolution of the dimensional changes throughout the curing process, measurements were taken at 2, 7, 14, 28, 56, 72, and 90 days. In addition, the mixtures were arranged in two environments, in dry and wet chambers.

This section focuses on showing the mechanical capabilities and dimensional changes of mortar mixtures. In the mortar mixtures, the M-EcHy mixture in which 25% of cement was replaced with mixed recycled sand powder and biomass bottom ash, and at 7, 28, and 90 days, there was a drop in compressive strength of 7.3%, 3.4%, and 2.8%, respectively. It is observed that in the long term, the substitution of cement with pBBA increased the strength [8].

#### 3.2.1. Results of Dimensional Changes of Mortar Mixtures

**Figure 5:**
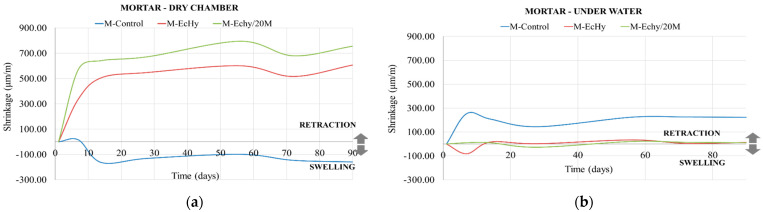
Dimensional changes for mortar mixtures. (**a**) Dry chamber environment; (**b**) under water environment.

In order to measure durability parameters, the dimensional changes of the mortar mixtures were studied. This parameter was measured for specimens subjected to two environments, in a humid chamber (20 °C and 70% RH) and submerged in water. Figure 5 shows the results, comparing the behaviour of the mixtures as a function of the environment to which they were subjected.

In the samples subjected to a dry chamber ambient, the dimensional changes in the control mortar showed low values. After 20 days of curing, there was volume stability, having a linear dimensional growth of 150 μm/m. Comparing the dimensional changes of the mixtures with recycled materials with respect to the control mix, it is observed that the volume stability was much lower, reaching a shrinkage of 600 μm/m for the M-EcHy mixture in which only cement was substituted and 750 μm/m for the M-Echy/20M mixture in which natural aggregates and cement were substituted.

In the samples submerged under water, the control mortar had greater dimensional changes than the recycled mortar mixtures, although volume stability was reached at 50 days for all samples. The stabilisation of the control mortar was reached with a shrinkage of 223 μm/m. The recycled mortar samples had a lower shrinkage than the control, being around 10 μm/m for both the M-EcHy and the M-Echy/20M mixtures.

#### 3.2.2. Results of Dimensional Changes in Concrete Mixtures

**Figure 6:**
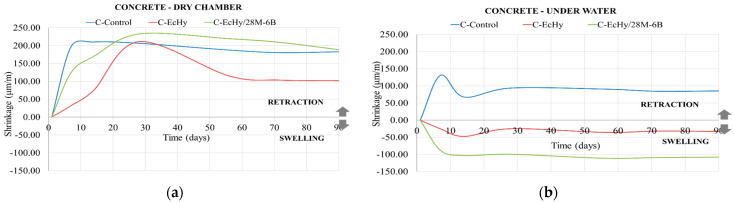
Dimensional changes for concrete mixtures. (**a**) Dry chamber environment; (**b**) under water environment.

As in the mortar mixtures, BBA has better long-term hardening (90 days). Results show that the increase in flexural strength from 7 days to 90 days is 25.4% for the C-EcHy/28M-6B mixture and 18.3% for the C-EcHy mix. For the C-Control mix, this increase in flexural strength is lower, being 14.7%. Dimensional change results are shown in Figure 6.

For the concrete subjected to the dry chamber, it is observed that the control mixture reached a volume stability at 14 days, with a shrinkage of 200 μm/m. For the mixtures with recycled materials, the volume stability was reached at 60 days, causing a shrinkage of 100 μm/m for C-EcHy and 200 μm/m for C-EcHy/28M-6B.

For concrete specimens subjected to a water-immersed environment, the volume stability of all specimens was reached at 25 days. The C-Control mixture decreased in size, having a final shrinkage of 85 μm/m. The mixtures with recycled elements had a swelling of 30 μm/m for C-EcHy and 110 μm/m for C-EcHy/28M-6B.

Mixtures with recycled elements showed similar dimensional change behaviour to conventional mixtures. Previous studies determined that, due to the saturation water demand of recycled aggregates, shrinkage increases [87]. There are determining factors that directly affect the dimensional changes in the mixes, such as the water–cement ratio or the setting time [88]. In addition, the lower density of the recycled aggregates (see Table 9 and Table 10, density and water absorption of recycled materials) and the porosity of the mortar added to the old recycled matrix also have repercussions for the dimensional changes in the concrete and mortar mixes [89,90].

## 4. LCA Methodology and Results

### 4.1. LCA Methodology

LCA is defined as the collection and evaluation of the inputs, outputs, and potential environmental impacts of a product system throughout its life cycle. According to the methodology established in the regulations ISO 14040 [91] and ISO 14044 [92], the application of the LCA consists of four stages: the definition of the objective and the scope, the analysis of the inventory, the evaluation of the impact of the life cycle, and finally, the interpretation of the results.

#### 4.1.1. LCA—Objective, Scope, and System Boundaries

In this study, LCA was carried out to evaluate the environmental impact derived from the use of MRA and BBA as a replacement for cement and/or natural aggregates in the manufacture of cement mortar and concrete as construction and building materials for concrete slabs in buildings. The dosages of the mortar and concrete mixtures to be evaluated are listed in Table 11 and Table 12.

**Figure 7:**
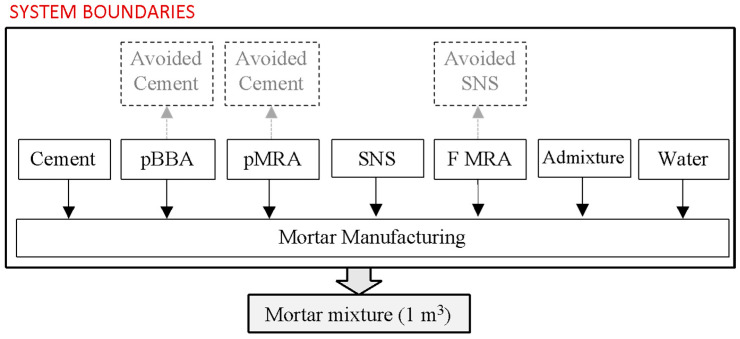
System boundaries of cement mortar production.

**Figure 8:**
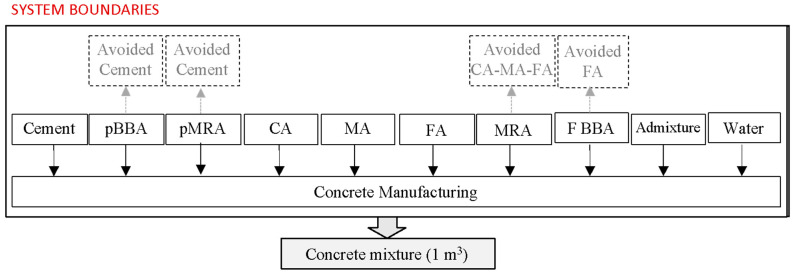
System boundaries of concrete production.

The functional unit in mortar and concrete mixtures corresponds to the manufacture of one cubic metre (1 m^3^). The system boundaries were established from cradle to gate, that is, at the product stage. The limits of the mortar and concrete system are shown in Figure 7 and Figure 8, respectively. Specifically, the system boundaries include the following:

- The manufacture of the components of the mortar and concrete mixtures;
- The avoided production of cement, since the use of pBBA and pMRA as a replacement for cement avoids the production of cement;
- Avoided production of natural aggregates. In the manufacture of mortar mixtures, the substitution of SNS for fine MRA avoids the production of SNS. In the manufacture of concrete, the incorporation of MRA and BBA prevents the production of CA, MA, and FA;
- As the materials to be used were available at the place of manufacture of the mortar and concrete mixtures, transport distances were not considered;
- The equipment required for the manufacturing process of mortar and concrete mixtures;

The energy consumption required by the equipment during the manufacturing process.

#### 4.1.2. Life-Cycle Inventory (LCI)

To generate the LCI, the main input and output flows of all the processes included in the system boundaries were compiled. The primary data correspond to specific data of the production processes collected at the place of production relative to the average production of the year 2021 provided by the producers. These data establish the energy consumption of the equipment, power, production, operation time, etc. For secondary data for materials, energy, and transportation, database processes were used from Ecoinvent v3.8 (allocation, cut-off by classification—unit “Cut-off, U”) [93].

The data used to generate the LCI are listed in Table 15. For the development of the inventory, the following considerations were taken into account:

- Inventories on the natural aggregates (CA, MA, FA, and SNS) were determined, according to the production processes, by means of primary data and the Ecoinvent v.3.8 database [93]. These aggregates were produced by a dolomitic quarry. The material was extracted using a bulldozer with a ripper without blasting and, after several crushing and screening processes, several fractions were obtained, including CA, MA, FA, and SNS.
- Inventory for the BBA was developed following the drying, sieving, and/or crushing treatment to which they were subjected, through the collection of primary data and the Ecoinvent v.3.8 database [93]. The treatment began when the wet ash was collected from the ashtray by means of a loader and transported to an outside storage area for air drying. Once BBA dried, it was collected in a storage area. To obtain 0/4 mm BBA, original BBA was moved to a screening area. The BBA grinding process was carried out in a ball mill for half an hour until the required fineness for pBBA was reached.
- MRA was produced in a C&DW treatment plant from non-selectively collected construction waste. The material, mostly from demolished buildings, was subjected to different processes of crushing, iron removal, blowing, and screening until different fractions of MRA were obtained. As there were practically no rejects, 100% of C&DW was recycled. From 1t of RCD treated, 0.28t of MRA (0/22 mm), 0.112 t of fine MRA (0/2 mm), and 0.608 t of other fractions were obtained. To obtain pMRA, MRA (0/22 mm) was run in a ball mill for three hours. The inventory of these components was generated from the primary data related to equipment and machinery, and with the processes of the Ecoinvent v.3.8 database [93];
- For the inventory of cement, water, and admixture, the processes of the Ecoinvent v.3.8 database [93] were used;
- For the mortar and concrete manufacturing process, “Concrete, high exacting requirement (CH), concrete production, Cut off, U” was used.

In order to comply with the data quality requirements related to technical, geographical, and technological representativeness, the Ecoinvent processes were modified according to the data provided by the producers. Likewise, electricity consumption was adapted to the process of the Spanish electricity network.

#### 4.1.3. Impact Assessment Methodology

According to the recommendations of the UNE-EN 15804:2012+A2 A2 [94] regarding sustainability of construction works, the impact assessment was conducted for the following categories: acidification (AP); climate change (GWP); eutrophication, freshwater (EP-freshwater); eutrophication, marine (EP-marine); eutrophication, terrestrial (EP-terrestrial); ozone depletion (ODP); photochemical ozone formation (POCP); resource use, fossils (ADP-fossils); resource use, minerals, and metals (ADP-min&met) and water use (WDP). For these categories, the characterisation factors recommended by the EC-JRC [95] were used. The data collected during the inventory phase were loaded into the SimaPro 9.4.0.49 software and processed using the EN 15804+A2 Method V1.02 [95]. The methodology of this impact assessment method was aligned with the EF method 3.0 published for use during the Environmental Footprint transition phase of the European Commission [95].

Initially, the impact values associated with the production of components were determined. Then, the impacts generated by the manufacture of cement mortar and concrete slab in building and concrete mixtures were calculated and compared in order to identify those that generated the greatest impact.

### 4.2. LCA Results and Discussion

#### 4.2.1. LCA of Component Materials

The impacts generated during the manufacture of 1 t of each component material of cement mortar and concrete mixtures are listed in Table 16. For the impact categories evaluated, the highest characterisation values correspond to the plasticiser admixture and second to CEM I. The impacts generated by the manufacture of CEM II were much higher than those generated during the processing of C&DW and BBA to obtain finely ground material, both pBBA and pMRA. The greatest variation occurred in the GWP category, since during the manufacture of CEM I, 869 kg CO_2_ eq. was emitted, while during the production of pBBA and pMRA, 1.25 kg CO_2_ eq./t and 4.89 kg CO_2_ eq./t were generated, respectively, which represents a reduction in emissions of up to 99.8%. Likewise, the manufacture of eco-hybrid cement made up of CEM I, pBBA, and pMRA generates 653 kg CO_2_ eq./t, which, compared to 799.9 kg CO_2_ eq./t emitted by CEM II, represents a reduction of 147 kg CO_2_ eq./t that is not released into the environment.

**Figure 9:**
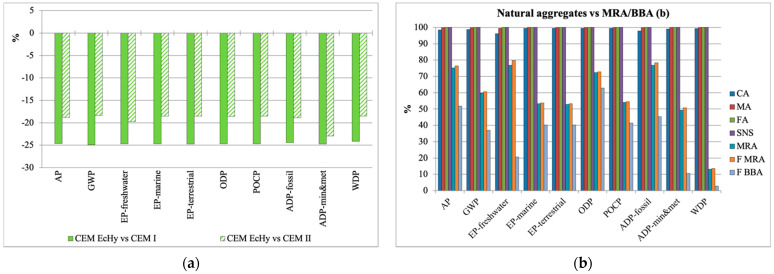
Comparative graph: CEM I 52.5/CEM II 43.5/CEM EcHy (**a**) and natural aggregates vs. MRA and BBA (**b**).

Analysing the three types of cement used in the study, CEM I reached the highest values and eco-hybrid cement the lowest. Comparatively, eco-hybrid cement loads were reduced in all impact categories, up to 25% compared to CEM I and up to 23% compared to CEM II (Figure 9a).

Regarding the impacts generated during the manufacture of natural and recycled aggregates (Figure 9b), the highest impacts corresponded to natural aggregates, specifically sand, since its manufacture requires more crushing and screening processes. In the case of recycled materials, the processing of C&DW to obtain MRA and fine MRA reduced environmental loads between 20% and 86% for the EP-freshwater and WDP categories, respectively. Likewise, the lowest impact values corresponded to fine BBA, which compared to natural sand (FA), for which characterisation values were reduced between 37% for ODP and 97% for WDP.

#### 4.2.2. LCA of Cement Mortar Mixtures

The characterisation values of the evaluated mortar mixtures are listed in Table 17. The control mortar reached the highest characterisation values in all impact categories, while the values were lower in the mortar mixtures made with recycled materials.

**Figure 10:**
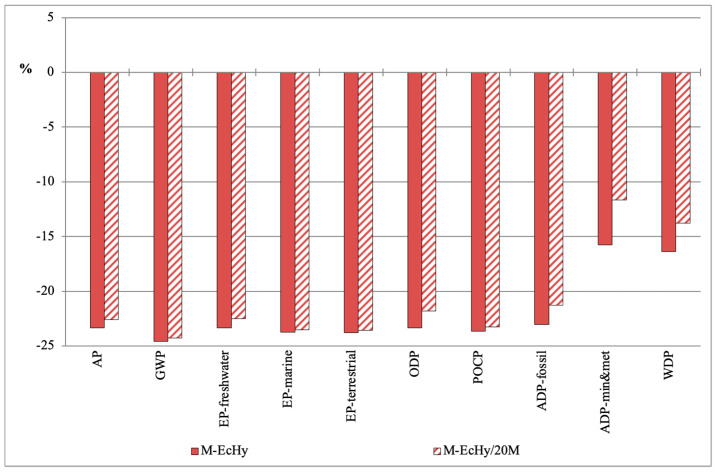
Variation in the characterisation values of the recycled mortar mixtures with respect to the control mortar.

Figure 10 shows comparatively the impact variations of the recycled mortar mixtures with respect to the control mortar. Specifically, the lowest impact values were generated in the M-EcHy mortar, since the replacement of cement with pBBA and pMRA caused reductions between 16% for the ADP-min&met category and 25% in the GWP category.

Likewise, in the M-EcHy/20M mortar, the impact reductions ranged between 12% for ADP-min&met and 24% for GWP (Figure 10). In this mortar, in order to achieve the adequate consistency with the replacement of FA with fine MRA, it was necessary to incorporate an admixture in the dosage, which made the impact values slightly higher than those of the M-EcHy mortar.

#### 4.2.3. LCA of Concrete Mixtures

The characterisation values of the concrete analysed (Table 18) show that the production of 1 m^3^ of C-Control generates 258.89 kg CO_2_ eq., while it is 229.43 kg CO_2_ eq. for C-EcHy and 233.71 kg CO_2_ eq. for C-EcHy/28M-6B. Also, the values for EP-freshwater and POF categories were lower in concrete with CEM EcHy.

**Figure 11:**
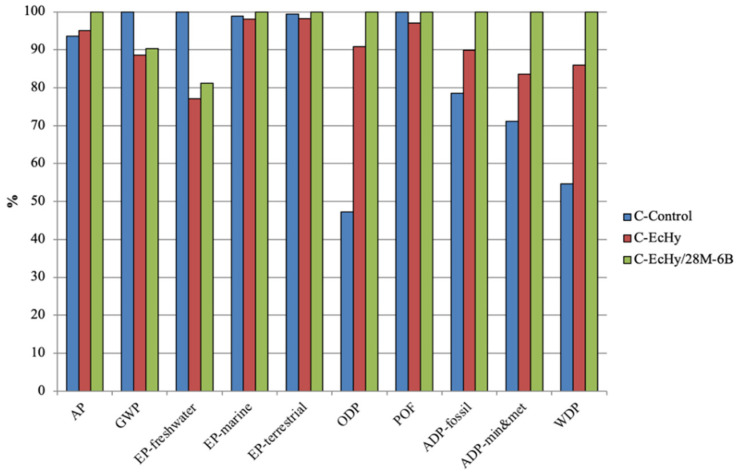
Comparative chart of characterisation values of concrete.

Figure 11 shows comparatively the impact variations in the concrete by categories. C-Control reached the highest values for GWP, EP-freshwater, and POF categories. For these categories, the loads were reduced in concrete mixtures with eco-hybrid cement, between 3% and 23% for C-EcHy and between 1% and 19% for C-EcHy/28M-6B. For the rest of the categories, the highest values corresponded to C-EcHy/28M-6B, for which values increased with respect to C-Control between 1% for EP-terrestrial and 53% for ODP. This increase was due to the replacement of natural aggregates (CA, MA, and FA) with fine BBA and MRA. These materials have a high water-absorption capacity, greater than natural aggregates, so to achieve adequate docility of the concrete, it was necessary to incorporate a plasticiser admixture and increase the amount of water in the dosage. For GWP, EP-freshwater, EP-marine, EP-terrestrial, and POF categories, the lowest values corresponded to C-EcHy concrete, as a consequence of the incorporation of eco-hybrid cement in concrete.

#### 4.2.4. LCA of Cement Mortar Mixtures vs. Concrete Mixtures

**Figure 12:**
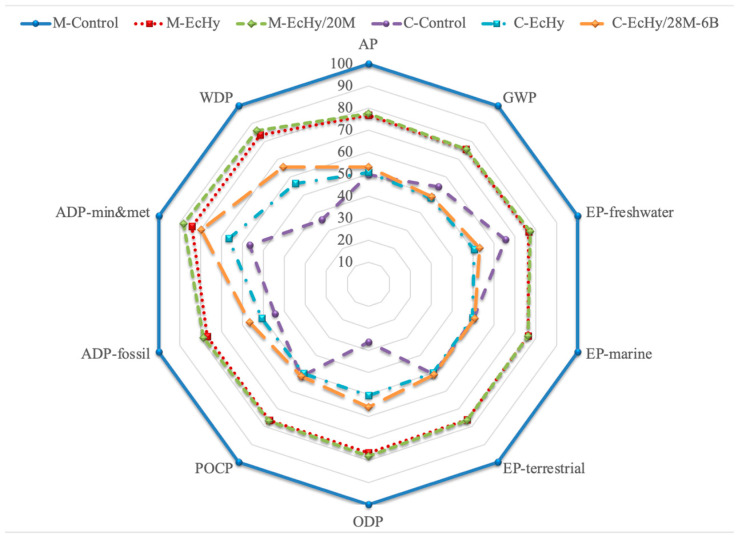
Comparative chart by impact categories of cement mortar and concrete.

Comparatively, the impacts associated with cement mortar and concrete mixtures are shown by categories in Figure 12. As can be seen, the M-Control mortar generated the highest impacts in all categories, so that the values of the rest of the mortar and concrete mixtures are shown relatively.

The lowest impacts in several categories (AP, ODP, ADP-fossil, ADP-min&met, and WDP) corresponded to C-Control, with reductions ranging from 43% for ADP-min&met to 73% for ODP. This decrease in loads compared to M-Control is directly associated with the lower amount of cement in the concrete dosage. For the other categories, concrete made with eco-hybrid cement, C-EcHy, presented the lowest impacts, with reductions of around 50% compared to M-Control.

According to the analysis carried out in Section 4.2.2 and Section 4.2.3, the partial replacement of cement with recycled materials (pBBA and pMRA) considerably reduced the environmental loads associated with cement mortar and concrete mixtures.

## 5. Environmental and Materials Costs: Methodology and Results

### 5.1. Environmental and Materials Costs Methodologies

Environmental and Materials Costs (EMC) is defined as the “economic assessment considering all agreed projected significant and relevant cost flows over a period of analysis expressed in monetary value. The projected costs are those needed to achieve defined levels of performance, including reliability, safety and availability” [96].

The European standard EN 60300-3-3 [97] serves as a guide to identify the elements that constitute the cost cycle and facilitates its management. This standard defines the EMC as “*the process of performing an economic analysis to assess the cost of an item over a portion, or all, of its life cycle in order to make decisions that will minimize the total cost of ownership while still meeting stakeholder requirements*”.

It is important to rule out that, as in the case of the EMC, the limits of the study can be established. The phases of a cost cycle analysis are the following: formulate the context and identify alternatives, define the scope and objectives of the analysis, identify constraints and relevant financial parameters, and define the analysis approach [96,97].

Environmental LCC is aligned with LCA in terms of system boundaries, functional units, inventory, and methodological steps. To assess the environmental cost, the Environmental PricesV1.02/European Environmental Prices (2015) [98] was adopted. This method developed by CE Delft is implemented in the SimaPro 9.4.0.49 software (PRé Sustainability, 2022). Specifically, it expresses the environmental impacts in monetary terms and indicates the loss of economic welfare that occurs when an additional kilogram of the pollutant reaches the environment. These prices are called external costs and allow an economic value to be given to the environmental impact produced by the materials. Environmental prices are constructed prices for the social cost or pollution, expressed in Euros per kilogram pollutant.

The environmental costs associated with the production of materials (components, mortars, and concrete) were calculated. In addition, the inventory of substances was determined to identify those that contributed mainly to the generation of the environmental cost, applying a cut-off value of 1.5%.

#### 5.1.1. EMC—Objective, Scope, and System Boundaries

In the case of the EMC, the cost of the materials to be used to manufacture the mortar and concrete mixtures was obtained. The cost of the manufacturing processes, which were considered for the calculation of the life cycle (Stage A1), were analysed. The materials studied were the following: CA, MA, FA, MRA, pBBA, pMRA, and BBA. For these materials, the following cost structure was obtained: direct costs: machinery (Amortization, O&M, and Energy) and direct labour and indirect costs (calculated as a percentage of direct costs).

**Figure 13:**
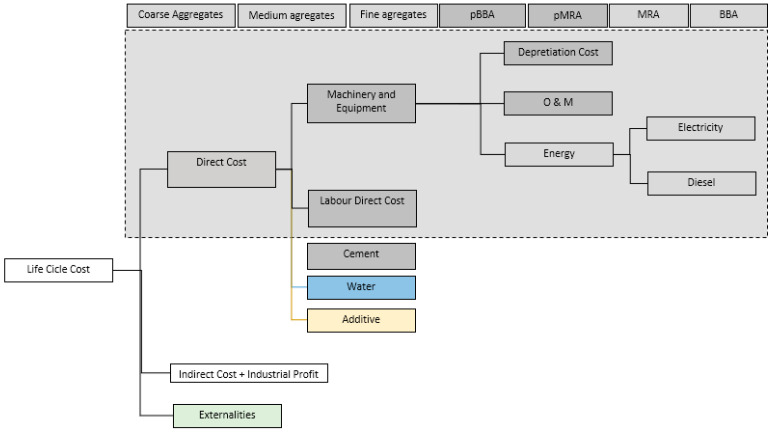
Cost structure of the materials analysed. Mortar and concrete mixtures.

The costs of materials, cement, water, and admixtures, were obtained directly from market values. Figure 13 represents the cost structure.

The objective was to calculate the variation in the cost of mortar and concrete mixtures when cement and aggregates were replaced with recycled materials. We use the estimated cost of the control mortar and concrete as a reference. The calculations were made for 1 m^3^, both for the case of mortar and concrete.

#### 5.1.2. Environmental and Materials Costs Inventory (EMCI)

For the cost breakdown structure concept, the processes considered for the calculation of the life cycle (Stage A1) are the ones that have been used to analyse the EMC. In the case of costs, there is no access to a specific construction and building materials price database that can be associated with SIMAPRO processes.

The authors programmed the processes that were taken into account for the manufacture of the materials using PYTHON language and following the cost structure indicated in the EN 60300-3-3 [97] standard and that applied to the case study object.

The structure costs considered (Stage A1) are as follows:

1. Direct costs.
  (a) Energy costs. Electricity (0.24 €/kWh). Diesel (1.34 €/l). The consumption of these inputs was calculated from the data of the production processes (Stage A1) that were previously defined. The values were obtained from SIMAPRO using the Ecoinvent database [93]. In order to comply with the data-quality requirements related to technical, geographical, and technological representativeness, the Ecoinvent processes were modified according to the data provided by the producers (see Section 4.1.2).
  (b) Calculation of the amortisation costs of the machinery. The calculation of the performance of the machinery (h/t) was obtained from the estimation of the time necessary to carry out a work cycle, taking into account the capacity of the machine and the different speeds of the activities carried out in the cycle. As an example, in the case of Handling by Shovel Loader, the loading time of the shovel bucket, the hauling distance with a load, and the unloaded return of the shovel were considered. In this case, a loader yield of 0.00125 h/t was obtained for a hauling distance of 130 m, which was the case of the gravel, sand, and cement manufacturing plant (located in Cordoba). This process was carried out to calculate the work cycles of the machinery necessary to manufacture each of the materials that were analysed.
  (c) Operation and maintenance (O&M) of machinery and equipment. For the calculation of the operation and maintenance costs, a useful life of the machinery of 10,000 h was considered. The depreciation cost was obtained by dividing the purchase price of the machinery by its useful life. O&M costs were 85% of amortisation costs and include, among others, tyres, overhauls, maintenance, and lubricant.
2. Indirect costs. Indirect costs were calculated as a percentage of direct costs. They include general expenses and industrial profit excluding transportation (Stage A2). From the results of the DELPHI methodology carried out with experts, a percentage of 40% of the direct costs was considered.

Cement costs (95.23 €/t), water (1.26 €/t), and admixture (123 €/t), considering the market costs.

From the prices of the materials, the manufacturing costs of the mortar and the concrete were calculated. Once the structure cost was calculated, the obtained prices were compared within the real market prices again using the DELPHI methodology, as indicated above.

Finally, the assessment of the externalities of the different mortar and concrete mixtures was included with the aim of taking into account the social costs and being able to obtain an economic assessment of each solution, as well as establish a series of conclusions.

### 5.2. EMC Results and Discussion

#### 5.2.1. EMC Results and Discussions of the Component Materials

**Figure 14:**
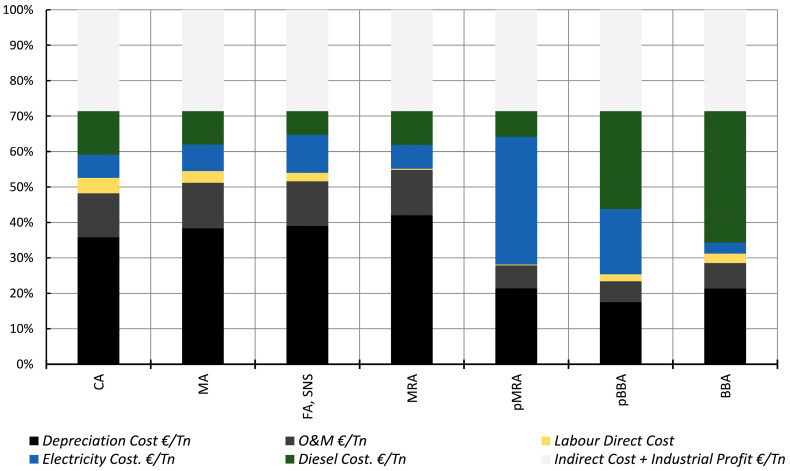
Mortar mixtures. Comparison chart of the costs being evaluated.

Table 19 summarises the cost structure of the different analysed materials. The considered costs are shown in Figure 14.

The cost of one tonne of cement at market, which was considered in this study, is 95.3 €/t. This price contrasts with the prices of the recycled materials that replace a proportional part of the cement for the manufacture of mortar and concrete mixtures: pBBA (2.68 €/t) and pMRA (13.76 €/t).

Related to these costs, the price increase in the case of pMRA is due to the final crushing and that needed to increase the vibrating screen process to reduce the size of the material. That estimated for the electricity is 4.59 €/t and represents 92.6% of the total cost of electricity consumption (4.95 €/t).

Table 20 shows the breakdown of the eco-hybrid cement costs that add to a total of €96.61/t. The price per tonne of cement type CEM I 52.5 is 124 €/t [99]. If the price of the eco-hybrid cement is compared with another equivalent (CEM II) and that contains around 15% of additions (e.g., blast furnace slag), it is observed that the prices are very similar (Cem II 42.5, €95.23/t).

The environmental costs and substance contribution by tonne of component materials are listed in Table 21. The highest environmental cost was 407.46 €/t, which corresponds to the plasticiser admixture, followed by the three types of cement in this order: 122.97 €/t for CEM I, 114.14 €/t for CEM II, and 92.58 €/t for eco-hybrid cement. For natural aggregates (CA, MA, FA, and SNS), the environmental cost of each of them was 0.44 €/t, while for MRA, it was 0.35 €/t, 0.36 €/t for fine MRA, and for pMRA, it was 1.84 €/t. Regarding BBA 0/2, the environmental costs were 0.17 €/t, and for pBBA, it was 0.43 €/t. These environmental costs were mainly due to air emissions of carbon dioxide-fossil, nitrogen oxides, and sulphur dioxide. In the case of natural aggregates and BBA, the emissions into the air of particulates lower than 2.5 µm was also relevant, as well as the land occupation of the C&DW treatment plant for the manufacture of MRA.

#### 5.2.2. EMC Results and Discussions: Cement Mortar Mixtures

The cost structure of the different mortar mixtures is listed in Table 22. The costs of the different mortar mixtures (EMC-mat) were compared with the reference mortar, M-Control. The estimated cost of the control mortar materials (cement, sand, and water) is 86.63 €/m^3^.

The costs of the materials of the mortar mixtures that replace part of the aggregates and cement with recycled aggregates and biomass ash are compared. The objective is to evaluate the impact of the substitutions with recycled material on the price of the materials. As in the case of the environmental impact analysed in LCA, cement cost represents the highest percentage (77.17%) of the total cost of the mortar materials.

**Figure 15:**
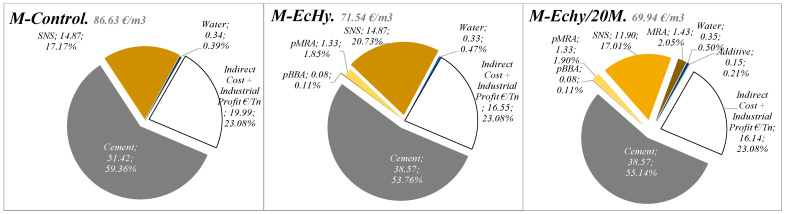
Mortar materials cost. Indirect and industrial and profit cost.

Figure 15 shows the costs of the mortar materials: M-Control, M-EcHy, and M-Echy/20M. The substitution of natural aggregates and cement with recycled material decreases the price of the mortar. In the case of the M-Echy/20M, it represents a decrease in the cost associated with materials of 17.4% (15.09 €/m^3^). In the case of the M-Echy/20M, the decrease is 19.2%. The decrease in price is mainly due to the cement. This is the most expensive material and represents 51.42% of the consumption. It must be considered that the price of cement tons is 95.23 €/t compared to 2.68 €/t and 13.77 €/t for pBBA and pMRA.

**Figure 16:**
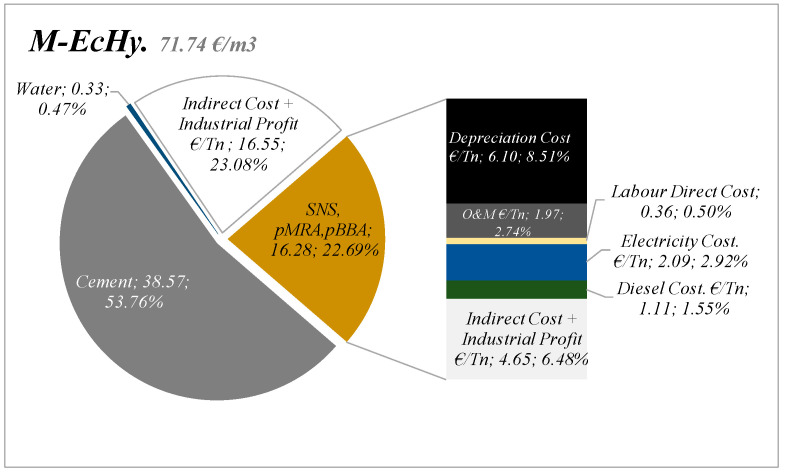
Characterisation of mortar mixture M-EcHy cost.

Figure 16 shows the cost structure of the materials that have been analysed, in the case of M-EcHy. The costs due to the energy consumption of recycled materials represented 2.92% of direct costs compared to 11.25% associated with equipment and machinery or 0.50% that direct labour implies.

If we add to these costs, the costs of transporting the materials to the factory, manufacturing of the dry mortar, and indirect costs, the price of the M-EcHy mortar would be 71.74 €/m^3^ compared to a cost of a control mortar of 86.63 €/m^3^.

Table 23 shows the environmental costs associated with cement mortar mixtures, as well as the substances that contribute more than 1%. As it can be seen, M-Control generated the highest environmental cost of 69.57 €/m^3^, while the cost of M-EcHy mortar was 53.16 €/m^3^ and that of the M-EcHy/20M was 53.61 €/m^3^. The incorporation of pBBA and pMRA as a replacement for cement reduced the environmental cost by 24%, and if SNS was also replaced with fine MRA, the cost reduction was 23%. Regarding the distribution of the environmental cost, the manufacturing stage of the components constitutes the main contribution, between 94% and 97%, and the mortar manufacturing would be responsible for around 3–6%. Mainly, the substances responsible for these environmental costs were the air emissions of carbon dioxide-fossil, nitrogen oxides, and sulphur dioxide.

#### 5.2.3. EMC Results and Discussions: Concrete Mixtures

**Figure 17:**
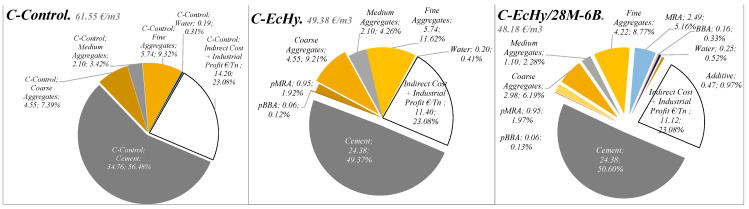
Concrete materials cost. Indirect and industrial and profit cost.

**Figure 18:**
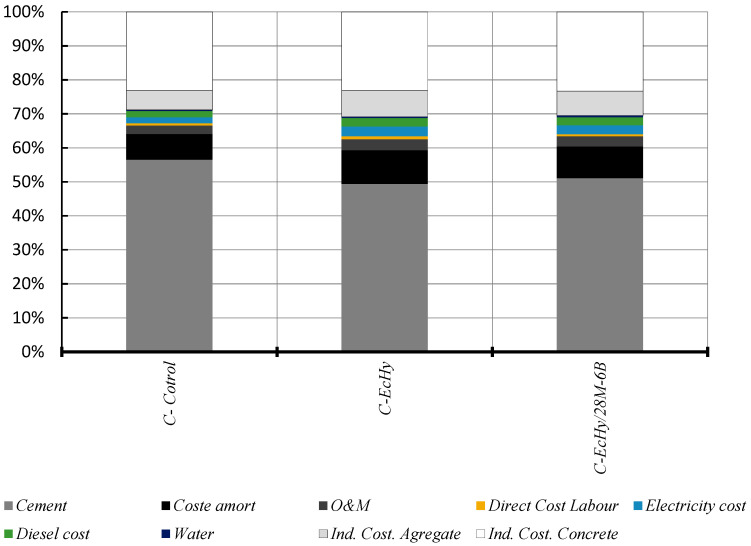
Concrete. Comparison chart of the costs being evaluated.

The distribution of the costs for the different concrete mixtures in relation to the materials that make them up is summarised in Figure 17 and Figure 18, and Table 24. It is shown that cement represents 73.42% in the case of C-Control concrete compared to 64.18% in the case of concrete mixtures that are manufactured with recycled material (C-Echy) or 65.78% in the case of C-EcHy/28M-6B. The use of recycled aggregates that replace natural aggregates has a lower impact on cost reduction.

Results show that cement represents 77.79% in the case of C-Control concrete compared to 70% in the case of concrete mixtures that are manufactured with recycled material. The use of recycled aggregates that replace natural aggregates has a lower impact on cost reduction because of 315 kg/m^3^ of CA (4.97 €/t), 155 kg/m^3^ of MA (6.47 €/t), and 165 kg/m^3^ of FA (6.56 €/t) for 472 kg/m^3^ of MRA (5.27 €/t) and 8 kg/m^3^ of BBA (2.00 €/t) for the case of concrete C- EcHy/28M-6B.

**Figure 19:**
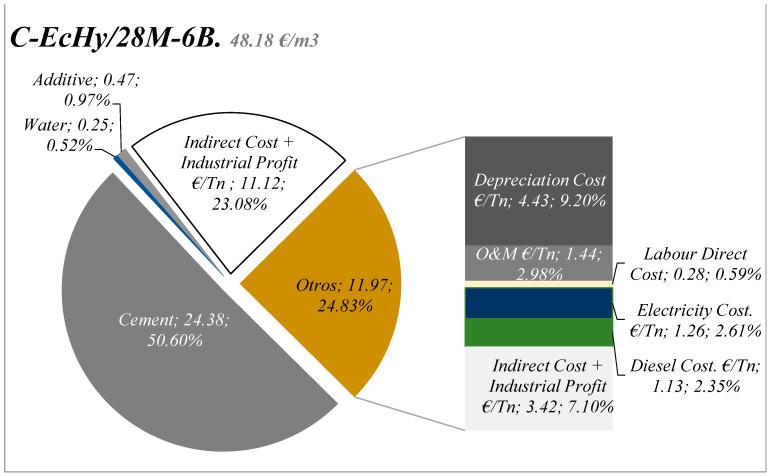
Characterisation of concrete C-EcHy/28M-6B cost.

The environmental costs associated with concrete slabs in building are listed in Table 25, as well as the substances that have a contribution equal to or greater than 1%. The results determine an environmental cost of 36.48 €/m^3^ for C-Control, 34.88 €/m^3^ for C-EcHy, and 36.33 €/m^3^ for C-EcHy/28M-6B, show in Figure 19 According to these values, the partial replacement of cement with pBBA and pMRA in concrete reduced the environmental cost by 4.38%, and 0.4% if the natural aggregates were also partially replaced by MRA and BBA. Regarding the distribution by stages, up to 93% of the environmental cost was due to the manufacture of the components, and around 7% was related to the concrete manufacturing process. Air emissions of carbon dioxide-fossil, nitrogen oxides, and sulphur dioxide constituted the main substances that generated environmental costs.

### 5.3. EMC Final Results

Comparatively, the total EMCs associated with cement mortar and concrete mixtures are shown in Table 26. As can be seen, the lowest total cost belongs to C-EcHy concrete, 84.26 Euros/m^3^, which compared to 98.03 euros/m^3^ of C-Control corresponds to a 14% reduction in cost.

In the case of mortars, the lowest cost is 123.55 Euros/m^3^ for the M-EcHy-20M mortar, resulting in a 21% cost reduction compared to 156.20 Euros/m^3^ for the M-Control.

**Figure 20:**
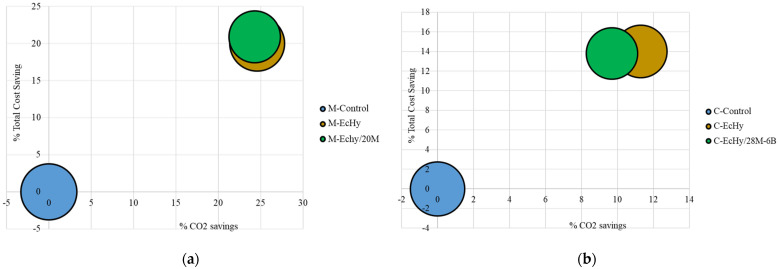
Correlation among optimal cost, CO_2_ savings, and mechanical performance. (**a**) Mortar mixtures. (**b**) Concrete mixtures.

A compilation of all of the final results of CO_2_ emission savings, monetary savings, and mechanical performance is presented in Figure 20. The *X*-axis shows the % reduction in CO_2_ emissions, the *Y*-axis shows the % economic savings, and the area of the circles determines the compressive strength at 90 days.

In the comparison of mortar mixes (Figure 20a), it can be seen that the two mixes achieved very similar emission and cost reductions. In this case, since M-EcHy has higher strengths, the optimum mix is M-EcHy. In the concrete mixes (Figure 20b), the compressive strengths were in the same range of magnitude. Looking at Figure 20b, the C-EcHy mix shows higher greenhouse gas emission reductions than the C-EcHy/28M-6B mix. Because of this, the optimum concrete mix is C-Echy.

## 6. Conclusions

The following conclusions can be extracted from the results obtained for the durability and mechanical strength of the mortar and concrete mixtures.

Concrete and mortar mixtures with eco-hybrid cement, applying a 25% substitution of cement, show a small drop compared to conventional cement mixtures at 90 days, only 2.8% for mortar mixtures and 6.3% for concrete mixtures in compressive strength terms. For earlier ages, the drop in mechanical strength is greater, with the hardening under hydration of the recycled phases being slower.

When natural aggregates are replaced with mixed recycled aggregates or biomass bottom ash, the drops are greater. The C-EcHy/28M-6B mixture has a 10% drop compared to C-Control. The M-EcHy/20M mixture has a 15% drop compared to M-Control. The BBA, in the long term, has considerable hardening, either substituting cement or substituting sand.

With respect to dimensional changes, the C-EcHy/28M-6B mixture behaves similarly to the control concrete mix. If we consider a slight drop in mechanical strength and similarity in terms of dimensional changes, the C-EcHy/28M-6B mixture is the most suitable.

The following conclusions can be drawn related to the LCA.

Regarding the impacts associated with the component materials, admixture and cement generate the highest values. The environmental burdens associated to eco-hybrid cement manufactured with filler materials derived from MRA and BBA are reduced by up to 23% compared to CEM II. As for MRA, the loads generated are reduced between 20% and 86% compared to natural aggregates, while obtaining fine BBA causes reduced impacts between 37% and 97% compared to natural sand.

The greatest contribution to the impact of cement mortar mixtures corresponds to the cement extraction and production stage, so the incorporation of MRA and BBA as a replacement for cement reduces environmental loads, between 16% and 25%. In addition, if natural sand is replaced by fine MRA, the environmental impacts are reduced between 12% and 24%.

Regarding the incorporation of eco-hybrid cement in concrete, the impact values are reduced between 3% and 23% for GWP, EP-freshwater, and POF categories. However, when natural aggregates are also replaced with BBA and MRA, the loads are increased between 1% for EP-terrestrial and 53% for ODP, because it is necessary to incorporate an admixture and increase the water dosage to provide the concrete with the required docility.

Regarding EMC, the following conclusions can be drawn.

The greatest impact of the cost of materials in the case of mortar and concrete mixtures is due to the consumption of cement. The reduction in the consumption of cement as a consequence of the substitution with recycled material allows the cost of materials to be reduced. In the case of mortar mixtures, the reduction is up to 20% and in the case of concrete mixtures, it is up to 22%, related to the cost of the mortar and the concrete control, respectively. The decrease in the cost impact of cement is related to the presented decrease based on the environmental impact

The environmental costs of the plasticiser admixture and cement are the highest of all the component materials. For pBBA and pMRA, environmental costs are reduced by up to 99.5% compared to the costs associated with cement. In this way, the partial substitution of cement with these recycled materials decreases the environmental costs associated with mortar and concrete mixtures by up to 24% and 4%, respectively.

Likewise, environmental costs decrease for MRA, up to 20%, and for BBA, up to 62%, with respect to natural aggregates. Therefore, the incorporation of these recycled aggregates as a partial substitution of natural aggregates reduces the environmental costs related to mortar and concrete mixtures by up to 23% and 0.4%, respectively.

These environmental costs are mainly generated by the air emissions of carbon dioxide-fossil, nitrogen oxides, and sulphur dioxide during the manufacturing stage of the component materials.

Regarding the contribution of the product stages, the material manufacturing generates the highest environmental costs, followed by the mortar or concrete manufacturing process.

## Figures and Tables

**Table 1:** Physicochemical properties of Cement CEM I 52.5R.

XRF (%)										UNE-EN 196-6 [52]
CEM I 52.5R	CaO	SiO_2_	SO_3_	Al_2_O_3_	Fe_2_O_3_	MgO	K_2_O	Na_2_O	TiO_2_	Density (kg/m^3^)
	70.03	17.06	4.49	3.63	1.89	1.36	0.94	0.29	0.31	3070

**Table 2:** Physicochemical characterisation of BBA powder (pBBA).

	Cement Substitute Wastes—Characterisation of pBBA			
Properties	Size		Results	Test Method
Density-SSD (kg/m^3^)	0–0.125 mm		2840	UNE-EN 1097-6 [53]
Water Absorption (%)	0–0.125 mm		20.96	
Sulphates (% SO_4_)	0–0.125 mm	Acid	0.29	UNE-ISO 11048 [54]
		Water	0.29	
Chlorides	0–0.125 mm		0.21	UNE-EN 1744-1 [55]
Organic Content (%)	0–0.125 mm		2.57	UNE 103204 [56]
Composition (%)	0–0.125 mm	SiO_2_ (%)	33.96	XRF
		CaO (%)	31.73	
		Al_2_O_3_ (%)	6.66	
		Fe_2_O_3_ (%)	2.94	
		MgO (%)	5.11	
		K_2_O (%)	15.32	
		SO_3_ (%)	0.57	
		Na_2_O (%)	0.31	
		TiO_2_ (%)	0.29	
		P_2_O_5_ (%)	2.97	
		MnO_2_ (%)	0.14	

**Table 3:** Physicochemical characterisation of MRA powder (pMRA).

	Cement Substitute Wastes—Characterisation of MRA Powder			
Properties	Size		Results	Test Method
Density-SSD (kg/m^3^)	0–0.125 mm		2910	UNE-EN 1097-6 [53]
Water Absorption (%)	0–0.125 mm		9.01	
Sulphates (% SO_4_)	0–0.125 mm	Acid	0.70	UNE-ISO 11048 [54]
		Water	0.24	
Chlorides (%)	0–0.125 mm		0.07	UNE-EN 1744-1 [55]
Organic Content (%)	0–0.125 mm		0.19	UNE 103204 [56]
Composition (%)	0–0.125 mm	SiO_2_ (%)	45.68	XRF
		CaO (%)	33.01	
		Al_2_O_3_ (%)	9.92	
		Fe_2_O_3_ (%)	3.38	
		MgO (%)	2.83	
		K_2_O (%)	1.93	
		SO_3_ (%)	1.59	
		Na_2_O (%)	0.68	
		TiO_2_ (%)	0.62	
		P_2_O_5_ (%)	0.19	
		MnO_2_ (%)	0.17	

**Table 4:** Particle size distribution.

Sieve (mm)	Lower Limit	Interval Average	Upper Limit
2	0	0	0
1.6	2	7	12
1	28	33	38
0.5	62	67	72
0.16	82	87	92
0.08	98	99	100

**Table 5:** Gross composition of MRA.

Constituents Composition MRA (0–22 mm) UNE-EN 933-11 [61]						
Concrete (%)	Natural Aggregates (%)	Ceramic (%)	Bituminous (%)	Others (%)	Glass (%)	Floating (cm^3^/kg)
44.0	34.5	18.6	2.3	0.6	0	0

**Table 6:** Physicochemical properties of MRA.

Properties	Characterisation of Fine Fraction MRA		
	Size	Results	Test Method
Density-SSD (kg/m^3^)	0–4 mm	2370	UNE-EN 1097-6 [53]
Water Absorption (%)	0–4 mm	9.42	
Friability Ratio (%)	0.1–2 mm	23.9	UNE 146404 [62]
Sand Equivalent (%)	0–2 mm	75.28	UNE-EN 933-8 [63]
Organic Content (%)	0–2 mm	0.19	UNE 103204 [56]
Chlorides (%)	0–2 mm	0.04	UNE-EN 1744-1 [55]
Water-soluble sulphates (%)	0–2 mm	0.35	
Acid-soluble sulphates (%)	0–2 mm	0.35	

**Table 7:** Physicochemical properties of Cement CEM II 42.5.

XRF (%)										UNE-EN 196-6 [52]
CEM II 42.5	CaO	SiO_2_	SO_3_	Al_2_O_3_	Fe_2_O_3_	MgO	K_2_O	Na_2_O	TiO_2_	Density (kg/m^3^)
	59.53	27.65	3.73	4.52	1.42	1.31	1.29	0.28	0.27	3110

**Table 8:** Physical characterisation of natural aggregates.

Properties	Characterisation of Natural Aggregate		
	Size	Results	Test Method
Density-SSD (kg/m^3^)	0–4 mm	2590	UNE-EN 1097-6 [53]
Water Absorption (%)		0.72	
Density-SSD (kg/m^3^)	4–12 mm	2640	
Water Absorption (%)		0.47	
Density-SSD (kg/m^3^)	12–22 mm	2660	
Water Absorption (%)		0.44	
Friability Ratio (%)	0.1–2 mm	14.8	UNE 146404 [62]
Sand Equivalent (%)	0–2 mm	87.7	UNE-EN 933-8 [63]
Crushing Value (%)	10–12.5 mm	18.58	ISO 20290-3 [68]
Los Angeles (%)	10–14 mm	20.0	UNE-EN 1097-2 [69]

**Table 9:** Physical characterisation of mixed recycled aggregates (MRA).

Properties	Characterisation of MRA		
	Size	Results	Test Method
Density-SSD (kg/m^3^)	0–4 mm	2370	UNE-EN 1097-6 [53]
Water Absorption (%)		9.42	
Density-SSD (kg/m^3^)	4–22 mm	2320	
Water Absorption (%)		6.49	
Friability Ratio (%)	0.1–2 mm	23.9	UNE 146404 [62]
Sand Equivalent (%)	0–2 mm	75.28	UNE-EN 933-8 [63]
Crushing Value (%)	10–12.5 mm	26.69	ISO 20290-3 [68]
Los Angeles (%)	10–14 mm	34.72	UNE-EN 1097-2 [69]

**Table 10:** Physicochemical properties of biomass bottom ash (BBA).

Properties	Characterisation of BBA		
	Size	Results	Test Method
Density-SSD (k)	0–4 mm	1.94	UNE-EN 1097-6 [53]
Water Absorption (%)	0–4 mm	19.82	
Friability Ratio (%)	0.1–2 mm	20.0	UNE 146404 [62]
Sand Equivalent (%)	0–2 mm	68.73	UNE-EN 933-8 [63]
Organic Content (%)	0–2 mm	3.1	UNE 103204 [56]
Chlorides (%)	0–2 mm	0.23	UNE-EN 1744-1 [55]
Water-soluble sulphates (%)	0–2 mm	0.29	
Acid-soluble sulphates (%)	0–2 mm	0.29	

**Table 11:** Dosages of mortar mixtures (kg/m^3^).

Series Name	Description		Dosages—Mortar Mixtures (kg/m^3^)							
		CEM I 52.5	CEMII 42.5	pBBA	pMRA	SNS (0/2 mm)	Fine MRA (0/2 mm)	Water	Water Absorption	Admixture
M-Control	Control CEM II 42.5	-	540	0	0	1620	0	270	0	0
M-EcHy	6% pBBA 19% pMRA	405	-	29.7	96.3	1620	0	265.5	0	0
M-EcHy/20M	6% pBBA 19% pMRA 20% MRA	405	-	29.7	96.3	1296	271.9	265.5	11.5	1.2

**Table 12:** Dosages of concrete mixtures.

Series Name	Description	Dosages—Concrete Mixtures (kg/m^3^)											
		CEM I 52.5	CEM II 42.5	pBBA	pMRA	CA (12/22 mm)	MA (4/12 mm)	FA (0/4 mm)	MRA (0/22 mm)	BBA (0/4 mm)	Water	Water Absorption	Admixture
C-Control	Control CEM II 42.5	-	365	0	0	915	325	625	0	0	153.3	0	0
C-EcHy	19% pMRA 6% pBBA	256	-	23	69	915	325	625	0	0	146.3	16	0
C-EcHy/28M-6B	19% pMRA 6% pBBA 28.5% MRA 6% BBA	256	-	23	69	600	170	460	472	80	146.3	53	3.8

**Table 13:** Mechanical strength for mortar mixtures.

Serie	Name	Description	Compressive S. (Mpa)						Flexural S. (Mpa)					
			7 Days		28 Days		90 Days		7 Days		28 Days		90 Days	
			μ	σ	μ	σ	μ	σ	μ	σ	μ	σ	μ	σ
Mortar	M-Control	Control CEM II 42.5	48.34	1.07	51.48	0.94	56.66	0.91	6.98	0.14	8.72	0.18	8.91	0.22
	M-EcHy	19% pMRA 6% pBBA	44.83	1.18	49.74	0.97	55.04	1.08	6.14	0.21	7.99	0.19	8.67	0.24
	M-Echy/20M	19% pMRA 6% pBBA 20% MRA	37.46	0.79	43.85	0.86	48.61	0.84	4.76	0.24	6.37	0.18	7.84	0.19

**Table 14:** Mechanical strength for concrete mixtures.

Serie	Name	Description	Compressive S. (Mpa)						Flexural S. (Mpa)					
			7 Days		28 Days		90 Days		7 Days		28 Days		90 Days	
			μ	σ	μ	σ	μ	σ	μ	σ	μ	σ	μ	σ
Concrete	C-Control	Control CEM II 42.5	49.7	1.23	55.7	1.34	58.4	1.09	5.09	0.19	5.68	0.18	5.84	0.22
	C-EcHy	19% pMRA 6% pBBA	44.5	1.01	49.1	1.14	54.7	1.12	4.81	0.14	5.38	0.16	5.74	0.19
	C-EcHy/28M-6B	19% pMRA 6% pBBA 28% MRA 6% BBA	39.4	0.98	43.0	1.01	52.3	1.08	4.32	0.21	4.56	0.18	5.42	0.17

**Table 15:** Characteristics of the equipment and processes used in the manufacture of cement mortar and concrete mixtures.

Materials	Process	Equipment	Amount	Power	Production	Operation Time	Electrical Consumption	Distance
				(kW)	(t/h)	(h)	(kWh/t)	(km)
Cement								
CEM I 52.5	Cement, Portland (Europe without Switzerland), production, Cut off, U, (Ecoinvent v3.8).							
CEM II 42.5	Cement, alternative constituents 6–20% (Europe without Switzerland) production, Cut off, U, (Ecoinvent v3.8).							
CEM EcHy	Cement, Portland (Europe without Switzerland), production, Cut off, U, (Ecoinvent v3.8).							
	pBBA							
	pMRA							
Natural aggregates								
CA 12/22 mm	Extraction	Bulldozer with ripper	1	-	998.87	-	-	-
MA 4/12 mm	Handling	Shovel loader	2	-	30.52	-	-	-
FA 0/4 mm	Handling	Conveyor belt, 25 m	1	20	168.92	-	0.118	-
SNS 0/2 mm		Conveyor belt, 15 m	2	8	112.61	-	0.071	-
		Conveyor belt, 5 m	2	4	123.87	-	0.032	-
	Screening	Vibrating screen	4	18.5	225	-	0.082	-
	Crushing	Impact mill	1	125.1	400	-	0.313	-
		Jaw crusher	1	206.1	400	-	0.515	-
Biomass Bottom Ash								
Fine BBA 0/2 mm	Handling	Shovel loader	4	-	31.221	-	-	-
pBBA	Transport	Shovel loader	2	-	-	0.006	-	0.03
	Transport	Shovel loader	2	-		0.01	-	0.05
	Screening	Vibrating screen	1	22.08	250	-	0.0883	-
	Crushing	Ball mill	1	15	4	0.5	1.88	-
	Handling	Conveyor belt, 5 m	1	4	22.55	-	0.2130	-
		Conveyor belt, 5 m	1	4	22.55	-	0.1770	-
Mixed Recycled Aggregate								
MRA 0/22 mm	Handling	Shovel loader	2	-	100	-	-	-
Fine MRA 0/2 mm	Transport	Shovel loader	1	-	-	0.02	-	0.1
pMRA	Transport	Shovel loader	1	-	-	0.01	-	0.05
	Handling	Overband	2	3.68	108.91	-	0.0338	-
		Blower	1	14	144.73	-	0.0967	-
		Vibrating plate	1	3	80		0.0375	-
		Conveyor belt, 15 m	1	7.36	148.51	-	0.0496	-
		Conveyor belt, 10 m	1	7.36	108.91	-	0.0676	-
		Conveyor belt, 5 m	5	4	108.91	-	0.0367	-
	Screening	Vibrating screen	4	22.08	250	-	0.0883	-
	Crushing	Jaw crusher	1	160	325	-	0.4920	-
		Impact mill	1	75	250	-	0.3000	-
		Ball mill	1	15	4	3	11.3	-
Water	Tap water (Europe without Switzerland), tap water production, conventional treatment, Cut-off, U (Ecoinvent v3.8).							
Admixture	Plasticiser, for concrete, based on sulfonated melamine formaldehyde (GLO-ES), production, Cut-off, U, (Ecoinvent v3.8).							

**Table 16:** Characterisation results of component materials (1 t).

Material	Impact category (Unit)									
	AP	GWP	EP-Freshwater	EP-Marine	EP-Terrestrial	ODP	POCP	ADP-Fossil	ADP min & met	WDP
	mol H+ eq	kg CO_2_ eq	kg P eq	kg N eq	mol N eq	kg CFC11 eq	kg NMVOC eq	MJ	kg Sb eq	m^3^depriv.
CEM I 52.5	1.97	869.52	8.47 × 10^−2^	5.30 × 10^−1^	6.02	2.61 × 10^−5^	1.51	3.29 × 10^3^	1.28 × 10^−3^	57.2
CEM II 42.5	1.83	799.91	7.94 × 10^−2^	4.90 × 10^−1^	5.56	2.41 × 10^−5^	1.40	3.07 × 10^3^	1.25 × 10^−3^	53.2
CEM EcHy	1.49	653.08	6.38 × 10^−2^	3.99× 10^−1^	4.53	1.96 × 10^−5^	1.14	2.49 × 10^3^	9.62 × 10^−4^	43.4
pBBA	9.60 × 10^−3^	1.25	3.04 × 10^−4^	2.40× 10^−3^	2.57 × 10^−2^	1.53 × 10^−7^	7.23 × 10^−3^	23.1	7.69 × 10^−6^	4.80 × 10^−1^
pMRA	4.06× 10^−2^	4.89	1.50 × 10^−3^	8.30 × 10^−3^	8.77 × 10^−2^	4.37 × 10^−7^	2.41 × 10^−2^	1.03 × 10^2^	1.85 × 10^−5^	2.59
CA	1.02 × 10^−2^	1.05	1.60 × 10^−4^	3.68 × 10^−3^	4.02 × 10^−2^	1.76 × 10^−7^	1.10 × 10^−2^	17.1	5.37 × 10^−6^	1.48
MA	1.04 × 10^−2^	1.07	1.66 × 10^−4^	3.70 × 10^−3^	4.05 × 10^−2^	1.77 × 10^−7^	1.11 × 10^−2^	17.5	5.42 × 10^−6^	1.49
FA	1.04 × 10^−2^	1.07	1.67 × 10^−4^	3.70 × 10^−3^	4.05 × 10^−2^	1.77 × 10^−7^	1.11 × 10^−2^	17.5	5.43 × 10^−6^	1.49
SNS	1.04 × 10^−2^	1.07	1.67 × 10^−4^	3.70 × 10^−3^	4.05 × 10^−2^	1.77 × 10^−7^	1.11 × 10^−2^	17.5	5.43 × 10^−6^	1.49
MRA	6.20 × 10^−3^	8.05 × 10^−1^	1.28 × 10^−4^	1.97 × 10^−3^	2.14 × 10^−2^	1.28 × 10^−7^	5.99× 10^−3^	13.4	2.67 × 10^−6^	1.95 × 10^−1^
Fine MRA	6.30 × 10^−3^	8.18 × 10^−1^	1.33 × 10^−4^	1.99 × 10^−3^	2.15 × 10^−2^	1.29 × 10^−7^	6.04× 10^−3^	13.7	2.75 × 10^−6^	2.03 × 10^−1^
BBA 0/2	3.85 × 10^−3^	5.54 × 10^−1^	3.45 × 10^−5^	1.49× 10^−3^	1.63 × 10^−2^	1.11 × 10^−7^	4.60× 10^−3^	7.95	5.76 × 10^−7^	4.10 × 10^−2^
Admixture	8.64	1.19 × 10^3^	3.52 × 10^−1^	1.10	11.8	2.10 × 10^−4^	4.37	2.92 × 10^4^	3.78 × 10^−2^	9.41 × 10^2^

**Table 17:** Characterisation results of cement mortar (1 m^3^).

Impact Category	Unit	Cement Mortar		
		M-Control	M-EcHy	M-EcHy/20M
AP	mol H+ eq	1.12	0.86	0.87
GWP	kg CO_2_ eq	475.78	358.91	360.21
EP-freshwater	kg P eq	4.83 × 10^−2^	3.70 × 10^−2^	3.74 × 10^−2^
EP-marine	kg N eq	0.30	0.23	0.23
EP-terrestrial	mol N eq	3.38	2.58	2.58
ODP	kg CFC11 eq	1.49 × 10^−5^	1.14 × 10^−5^	1.16 × 10^−5^
POCP	kg NMVOC eq	0.86	0.65	0.67
ADP-fossil	MJ	1879.75	1446.38	1479.53
ADP-min&met	kg Sb eq	1.08 × 10^−3^	9.10 × 10^−4^	9.54 × 10^−4^
Water use	m^3^ depriv.	46.77	39.12	40.32

**Table 18:** Characterisation results of concrete mixtures (1 m^3^).

Impact Category	Unit	Concrete Mixtures		
		C-Control	C-EcHy	C-EcHy/28M-6B
AP	mol H+ eq	0.56	0.57	0.60
GWP	kg CO_2_ eq	258.89	229.43	233.71
EP-freshwater	kg P eq	3.16 × 10^−2^	2.44 × 10^−2^	2.57 × 10^−2^
EP-marine	kg N eq	0.15	0.15	0.15
EP-terrestrial	mol N eq	1.71	1.69	1.72
ODP	kg CFC11 eq	3.91 × 10^−6^	7.53 × 10^−6^	8.29 × 10^−6^
POCP	kg NMVOC eq	0.44	0.43	0.44
ADP-fossil	MJ	835.57	956.83	1063.94
ADP-min&met	kg Sb eq	6.13 × 10^−4^	7.20 × 10^−4^	8.62 × 10^−4^
Water use	m^3^ depriv.	16.82	26.42	30.74

**Table 19:** Summary of the cost structure of the different materials analysed.

Category Cost (€/tn)	pBBA	pMRA	CA	MA	FA	SNS	MRA	Fine MRA	BBA 0/2
Depreciation Cost	0.47046	2.94631	1.77992	2.48392	3.58392	3.58392	2.21895	2.21895	0.42646
O&M	0.15789	0.89506	0.61753	0.82873	1.15873	1.15873	0.67127	0.67127	0.14469
Labour Direct Cost	0.05224	0.03483	0.21657	0.21657	0.21657	0.21657	0.01741	0.01741	0.05224
Electricity cost	0.49378	4.95588	0.32476	0.48508	0.98742	0.98742	0.35707	0.35707	0.06377
Diesel cost	0.74128	1.00053	0.61134	0.61134	0.61134	0.61134	0.50027	0.50027	0.74128
Total Direct Cost	1.91564	9.83261	3.55012	4.62564	6.55798	6.55798	3.76497	3.76497	1.42844
Indirect Cost + Industrial Profit (IP)	0.76626	3.93304	1.42005	1.85026	2.62319	2.62319	1.50599	1.50599	0.57138
Price	2.68190	13.76565	4.97017	6.47590	9.18118	9.18118	5.27096	5.27096	1.99982

**Table 20:** Eco-hybrid cement costs.

Material			Quantity (Tn)	Price (€/Tn)	Total
Eco-hybrid cement	Cement CEM I		0.75	124.00	93.00
	pBBA		0.06	2.68	0.16
	pMRA		0.19	13.77	2.62
Proportional share of indirect costs and industrial profit of added materials pBBA, pMRA.			30.00%	0.83	0.83
					96.61 €/Tn

**Table 21:** Environmental cost of materials and substance contribution (1 t).

Substance/Compartment	Unit	CEM I 52.5	CEM II 42.5	CEM EcHy	pBBA	pMRA	CA	MA	FA	SNS	MRA	Fine MRA	BBA 0/2	Admixture
	Euro	122.97	114.14	92.58	0.43	1.84	0.44	0.44	0.44	0.44	0.35	0.36	0.17	407.46
Arsenic (air)	%	0.79	0.82	0.79	1.25	0.92	0.84	0.84	0.84	0.84	0.56	0.57	0.26	5.07
Arsenic (water)	%	1.20	1.23	1.21	2.56	3.01	1.33	1.36	1.36	1.36	1.29	1.32	0.65	3.46
Barium (water)	%	0.49	0.50	0.49	0.67	0.53	0.52	0.52	0.52	0.52	0.42	0.42	0.46	2.20
Cadmium (air)	%	0.18	0.19	0.18	0.23	0.14	0.17	0.17	0.17	0.17	0.11	0.11	0.06	1.10
Carbon dioxide, fossil (air)	%	38.81	38.46	38.72	15.11	13.83	12.91	12.92	12.93	12.93	12.21	12.23	17.85	13.71
Lead (air)	%	0.66	0.68	0.66	0.83	0.60	0.58	0.58	0.58	0.58	0.39	0.40	0.23	3.48
Manganese (water)	%	7.23	7.31	7.23	7.24	8.59	3.77	3.86	3.87	3.87	3.73	3.82	2.02	9.10
Methane, fossil (air)	%	0.59	0.59	0.59	0.67	0.73	0.42	0.43	0.43	0.43	0.41	0.41	0.38	2.28
Nitrogen oxides (air)	%	21.71	21.60	21.71	27.82	22.31	43.45	43.14	43.08	43.08	28.68	28.53	45.79	12.96
Particulates, <2.5 µm (air)	%	4.36	4.42	4.38	12.55	10.87	15.52	15.46	15.45	15.45	11.10	11.09	16.02	9.77
Particulates, >2.5 µm, and <10 µm (air)	%	5.59	5.72	5.58	3.55	2.53	2.84	2.85	2.85	2.85	2.25	2.27	2.48	4.76
Sulphur dioxide (air)	%	10.91	10.97	10.94	17.36	20.29	10.87	11.03	11.06	11.06	10.36	10.53	8.82	23.57
Remaining substances (air)	%	7.49	7.52	7.52	10.16	15.64	6.77	6.85	6.86	6.86	28.49	28.30	4.97	8.52

**Table 22:** Calculation of EMC-mat to be applied in mortar mixtures (1 m^3^).

Mortar	Category Cost (€/m^3^)	Cement	Water	Additive	pBBA	pMRA	SNS	MRA	Total
M-Control	Cost amort	-							
							5.806		5.806
	O&M	-					1.877		1.877
	Direct Cost Labour	-					0.351		0.351
	Electricity cost	-					1.600		1.600
	Diesel cost	-					0.990		0.990
	Total Indirect Cost Materials	-					4.250		4.250
	TOTAL COST	43.71	0.28				12.678		
	Total Indirect EMC-MAT Mortar (15%)								19.991
		51.424	0.340				14.874		**86.629**
M-EcHy		38.568	0.335						38.903
	Cost amort				0.014	0.284	5.806		6.104
	O&M				0.005	0.086	1.877		1.968
	Direct Cost Labour				0.002	0.003	0.351		0.356
	Electricity cost				0.015	0.477	1.600		2.092
	Diesel cost				0.022	0.096	0.990		1.109
	Total Indirect. Cost.				0.023	0.379	4.250		4.651
	Total Indirect Cost Mortar								16.554
		38.568	0.335		0.080	1.326	14.874		**71.736**
M-EcHy/20		38.568	0.349	0.148					39.065
	Cost amort				0.014	0.284	4.645	0.603	5.546
	O&M				0.005	0.086	1.502	0.183	1.775
	Direct Cost Labour				0.002	0.003	0.281	0.005	0.290
	Electricity cost				0.015	0.477	1.280	0.097	1.869
	Diesel cost				0.022	0.096	0.792	0.136	1.047
	Additive								
	Total Indirect Cost				0.023	0.379	3.400	0.409	4.211
	Total Indirect Cost Mortar								16.141
		38.568	0.349	0.148	0.080	1.326	11.899	1.433	**69.943**

Note: The amount highlighted in bold is the total cost for the manufacture of 1 m^3^ of each mortar mixture.

**Table 23:** Environmental cost of cement mortar mixtures (1 m^3^) and substance contribution.

EMC-ENV Stage		Substance/Compartment	M-Control		M-EcHy		M-EcHy/20M	
			Euro	%	Euro	%	Euro	%
Total cost			69.57	100	53.16	100	53.61	100
Materials			67.14	96.51	50.74	94.44	51.18	95.47
	CEM I 52.5		66.41	95.44	49.80	93.68	49.80	92.91
	pBBA		-	-	0.01	0.02	0.01	0.02
	pMRA		-	-	0.18	0.33	0.18	0.33
	SNS		0.72	1.04	0.72	1.36	0.58	1.08
	Fine MRA		-	-	-	-	0.10	0.18
	Water		0.02	0.03	0.02	0.04	0.02	0.04
	Admixture		-	-	-	-	0.49	0.91
Cement mortar manufacturing			2.43	3.49	2.43	4.56	2.43	4.53
		Arsenic (air)	0.92	1.32	0.64	1.20	0.74	1.38
		Arsenic (water)	0.77	1.10	0.72	1.36	0.66	1.23
		Carbon dioxide, fossil (air)	26.10	37.51	19.68	37.03	19.74	36.83
		Manganese (water)	5.08	7.30	3.89	7.32	3.94	7.34
		Mercury (air)	1.6	2.68	1.40	2.63	1.40	2.61
		Nitrogen oxides (air)	14.98	21.54	11.42	21.49	11.45	21,36
		Particulates, <2.5 µm (air)	3.19	4.58	2.49	4.68	2.52	4.71
		Particulates, >2.5 µm, and <10 µm (air)	3.88	5.58	2.96	5.57	2.98	5.56
		Sulphur dioxide (air)	7.75	11.14	5.98	11.25	6.09	11.36
		Remaining substances	5.04	7.25	3.98	7.48	4.08	7.61

**Table 24:** Calculation of EMC-mat to be applied in concrete mixtures (1 m^3^).

Concrete	Category Cost (€/m^3^)	Cement	Water	Addit	pBBA	pMRA	CA	MA	FA, SNS	MRA	BBA 0/2	Total
C- Control		34.75895	0.19316									34.95211
	Cost amort						1.62863	0.80727	2.23995			4.67586
	O&M						0.56504	0.26934	0.72421			1.55859
	Direct Cost Labour						0.19816	0.07039	0.13536			0.40390
	Electricity cost						0.29715	0.15765	0.61714			1.07194
	Diesel cost						0.55937	0.19868	0.38209			1.14015
	Ind. Cost. Aggregate						1.29934	0.60133	1.63950			3.54017
	Ind. Cost. Concrete											14.20281
		34.75895	0.19316				4.54770	2.10467	5.73823			**61.54553**
C-EcHy		24.37888	0.20450									24.58338
	Cost amort				0.01082	0.20330	1.62863	0.80727	2.23995			4.88997
	O&M				0.00363	0.06176	0.56504	0.26934	0.72421			1.62398
	Direct Cost Labour				0.00120	0.00240	0.19816	0.07039	0.13536			0.40751
	Electricity cost				0.01136	0.34196	0.29715	0.15765	0.61714			1.42525
	Diesel cost				0.01705	0.06904	0.55937	0.19868	0.38209			1.22623
	Ind. Cost. Aggregate				0.01762	0.27138	1.29934	0.60133	1.63950			3.82918
	Ind. Cost. Concrete											11.39565
		24.37888	0.20450		0.06168	0.94983	4.54770	2.10467	5.73823			**49.38115**
C-EcHy/28M-6B		24.37888	0.25112	0.46816								25.09816
	Cost amort				0.01082	0.20330	1.06795	0.42227	1.64860	1.04735	0.03412	4.43440
	O&M				0.00363	0.06176	0.37052	0.14088	0.53302	0.31684	0.01158	1.43823
	Direct Cost Labour				0.00120	0.00240	0.12994	0.03682	0.09962	0.00822	0.00418	0.28238
	Electricity cost				0.01136	0.34196	0.19485	0.08246	0.45421	0.16854	0.00510	1.25848
	Diesel cost				0.01705	0.06904	0.36680	0.10393	0.28122	0.23613	0.05930	1.13346
	Ind. Cost. Aggregate				0.01762	0.27138	0.85203	0.31454	1.20667	0.71083	0.04571	3.41878
	Ind. Cost. Concrete											11.11917
		24.37888	0.25112	0.46816	0.06168	0.94983	2.98210	1.10090	4.22334	2.48789	0.15999	**48.18306**

Note: The amount highlighted in bold is the total cost for the manufacture of 1 m^3^ of each concrete mixture.

**Table 25:** Environmental EMC-ENV cost of concrete (1 m^3^) and substance contribution.

EMC Stage		Substance/Compartment	C-Control		C-EcHy		C-EcHy/28M-6B	
			Euro	%	Euro	%	Euro	%
Total cost			36.48	100	34.88	100	36.33	100
Materials			34.05	93.35	32.45	93.04	33.91	93.32
	CEM II 42.5		33.22	91.06	-	-	-	-
	Eco-hybrid cement		-	-	31.62	90.64	31.62	87.02
		CEM I 52.5	-	-	31.48	90.25	31.48	86.65
		pBBA	-	-	0.01	0.03	0.01	0.03
		pMRA	-	-	0.13	0.36	0.13	0.35
	CA		0.40	1.10	0.40	1.15	0.26	0.72
	MA		0.14	0.40	0.14	0.41	0.08	0.21
	FA		0.28	0.76	0.28	0.80	0.20	0.56
	MRA		-	-	-	-	0.17	0.46
	Fine BBA		-	-	-	-	0.01	0.04
	Water		0.01	0.04	0.01	0.04	0.02	0.05
	Admixture		-	-	-	-	1.55	4.26
Concrete manufacturing			2.43	6.65	2.43	6.96	2.43	6.68
		Arsenic (air)	0.73	2.01	0.49	1.41	0.57	1.57
		Arsenic (water)	0.53	1.45	0.50	1.44	0.56	1.53
		Carbon dioxide, fossil (air)	13.65	37.42	12.58	36.06	12.78	35.16
		Carbon-14 (air)	0.24	0.67	0.21	0.60	0.22	0.59
		Manganese (water)	3.31	9.09	2.57	7.36	2.70	7.44
		Nitrogen oxides (air)	7.44	20.41	7.48	21.44	7.61	20.95
		Particulates, <2.5 µm (air)	1.39	3.82	1.70	4.87	1.83	5.03
		Particulates, >2.5 µm, and <10 µm (air)	2.52	6.91	1.94	5.55	2.01	5.52
		Radon-222 (air)	0.34	0.93	0.28	0.81	0.29	0.81
		Sulphur dioxide (air)	3.77	10.34	3.98	11.41	4.33	11.92
		Remaining substances	2.54	6.95	3.16	9.07	3.44	9.47

**Table 26:** Environmental and material total costs (EMCs) of concrete and mortar mixtures.

Mortar	Material Cost		Environmental Cost		Total Cost
	(Euros/m^3^)	(%)	(Euros/m^3^)	(%)	(Euros/m^3^)
M- Control	86.63	55.5	69.57	44.5	156.20
M-EcHy	71.74	57.4	53.16	42.6	124.90
M-EcHy/20M	69.94	56.6	53.61	43.4	123.55
C-Control	61.55	62.8	36.48	37.2	98.03
C-EcHy	49.38	58.6	34.88	41.4	84.26
C-EcHy/28M-6B	48.18	57.0	36.33	43.0	84.51

## Data Availability

The data is shown in the article.
